# Supplementary material for: Clinical evaluation of the Diagnostic Analyzer for Selective Hybridization (DASH): A point-of-care PCR test for rapid detection of SARS-CoV-2 infection
Source: PLoS One. 2022 Jun 16;17(6):e0270060. doi: 10.1371/journal.pone.0270060 (PMC9202852; doi:10.1371/journal.pone.0270060)
Supplement: S1 File — (PDF) [file pone.0270060.s002.pdf]

DASH by Minute Molecular

Diagnostic Analyzer for Specific Hybridization

DASH SARS-CoV-2/S Test | Instructions for Use

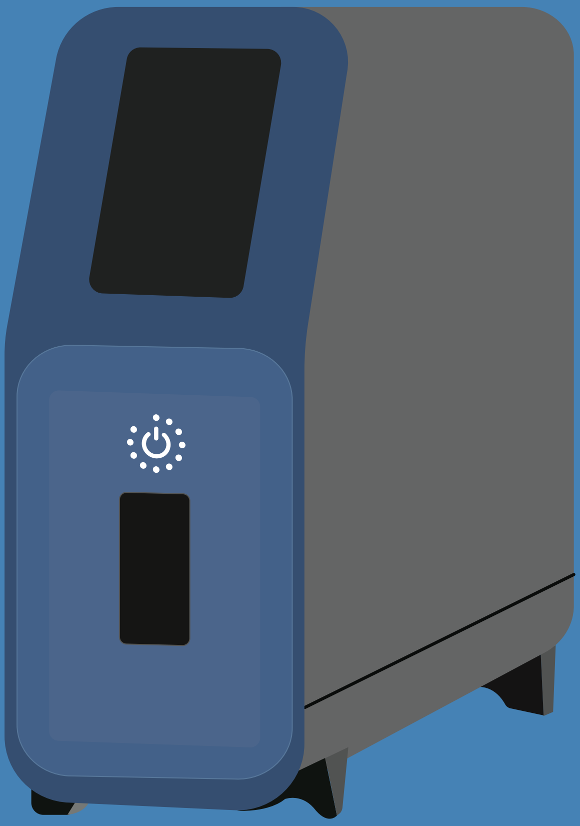

For Emergency Use Authorization (EUA) Only

For Direct Anterior Nasal Swab Specimens

REF IVD ℞ ONLY

PN-0205 | REV 2022-03-09

Minute Molecular Diagnostics, Inc.
1800 Sherman Avenue, Suite 504
Evanston, IL 60201, USA

1-888-992-DASH

www.m2dx.com

For questions, assistance, or if the DASH Analyzer or DASH SARS-CoV-2/S Test is not performing as expected, contact us at **techservice@m2dx.com**

Contents

Proprietary Name	3
Common or Usual Name	3
Intended Use	3
Summary and Explanation	3
Principle of the Procedure	3
Reagents and Analyzer	4
Materials Provided	4
Required But Not Provided	4
Storage and Handling	4
Warnings and Precautions	5
Chemical Hazards	5
Sample Collection, Transport and Handling	6
Quality Control - Internal Controls	6
Quality Control - External Controls	6
The DASH System	7
1. Unpacking Instructions	7
2. Installation and Set-Up	9
3. Running QC Samples	15
4. Running Patient Samples	18
Result Interpretation	22
Retests	23
5. Logging Out and Shutting Down	23
6. Editing Settings and Viewing Reports	24
Limitations	30
Conditions of Authorization for Laboratories	31
Performance Characteristics	32
Analytical Performance	32
Headquarters Location	38
Technical Assistance	38
References	38
Table of Symbols	38

Proprietary Name

DASH SARS-CoV-2/S Test

Common or Usual Name

DASH SARS-CoV-2/S Test

Intended Use

The DASH SARS-CoV-2/S Test is a real-time RT-PCR assay intended for the qualitative detection of RNA from SARS-CoV-2 in direct anterior nasal swab specimens from individuals suspected of COVID-19 by their healthcare provider. Testing is limited to laboratories certified under the Clinical Laboratory Improvement Amendments of 1988 (CLIA), 42 U.S.C. §263a, that meet the requirements to perform high, moderate, or waived complexity tests. The DASH SARS-CoV-2/S Test is authorized for use at the Point of Care (POC), i.e., in patient care settings operating under a CLIA Certificate of Waiver, Certificate of Compliance, or Certificate of Accreditation.

Results are for the identification of SARS-CoV-2 RNA. The SARS-CoV-2 RNA is generally detectable in anterior nasal specimens during the acute phase of infection. Positive results are indicative of the presence of SARS-CoV-2 RNA; clinical correlation with patient history and other diagnostic information is necessary to determine patient infection status. Positive results do not rule out bacterial infection or co-infection with other viruses. The agent detected may not be the definite cause of disease.

Negative results do not preclude SARS-CoV-2 infection and should not be used as the sole basis for patient management decisions. Negative results must be combined with clinical observations, patient history, and epidemiological information.

Laboratories within the United States and its territories are required to report all test results to the appropriate public health authorities.

The DASH SARS-CoV-2/S Test is intended for use by health professionals or trained operators who are proficient in using the DASH Analyzer. The DASH SARS-CoV-2/S Test is only for use under the Food and Drug Administration's Emergency Use Authorization.

Summary and Explanation

Coronavirus disease 2019 (COVID-19) is a potentially fatal respiratory illness caused by the novel human coronavirus SARS-CoV-2 (severe acute respiratory syndrome coronavirus 2) which has resulted in significant worldwide morbidity and mortality. In March 2020, The World Health Organization declared COVID-19 a global public health emergency.

DASH SARS-CoV-2/S Test uses reverse transcription-quantitative polymerase chain reaction (RT-qPCR) for rapid qualitative detection of SARS-CoV-2 from anterior nasal swabs. The DASH platform enables the performance of the DASH SARS-CoV-2/S Test at the point-of-care or in clinical laboratory settings.

Principle of the Procedure

The DASH SARS-CoV-2/S Test combines the technologies of sequence specific capture sample prep and RT-qPCR amplification. The DASH SARS-CoV-2/S Test Cartridge contains all reagents necessary to perform the test. An anterior nasal with a 30-mm breakpoint is used to collect a specimen. The nasal swab specimen is added to the DASH SARS-CoV-2/S Test Cartridge sample chamber containing lysis buffer which releases the RNA target from the virus. The operator breaks off the swab at the 30-mm breakpoint, and the Cartridge is capped and inserted into the DASH Analyzer to initiate the test. All subsequent test steps are performed automatically by the DASH Analyzer.

The target RNA molecules are isolated from specimens by use of capture oligomers via target capture that utilizes paramagnetic particles. The biotinylated capture oligomers contain sequences complementary to specific regions of the target molecules. During the hybridization step, the sequence specific regions of the capture oligomers bind to specific regions of the target molecules. The capture oligomer:target complex is then concentrated by the addition of streptavidin-coated paramagnetic particles. The paramagnetic particles including the bound target molecules are washed to remove residual specimen matrix that may contain amplification reaction inhibitors. After the target capture steps are completed, the target RNA is ready for amplification.

The RT-qPCR assay targets two regions of the Nucleocapsid protein (N) gene that are unique to SARS-CoV-2. The two regions are not differentiated, and the amplification of either one or both targets leads to fluorescent signal. A process control is also included to control for adequate processing of the target virus through steps of sample purification, nucleic acid amplification and to monitor for the presence of inhibitors in the RT-qPCR test.

Reagents and Analyzer

The **DASH SARS CoV-2/S Test** contains sufficient reagents to process 12 specimens or quality control samples.

Storage and Handling

Store the DASH SARS-CoV-2/S Test Cartridges and DASH Analyzer at 15-30˚C.

DO NOT open Cartridge Cover #1, until you are ready to perform testing.

DO NOT use a Cartridge that is wet or has leaked.

Materials Provided

Quick Reference Guide (QRG) and Instructions For Use (IFU) (not shown)

DASH SARS-CoV-2/S Cartridge

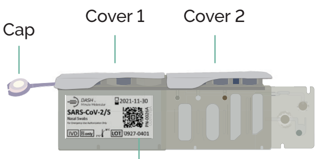

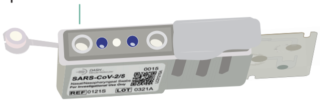

(Top View of Cartridge With Cover 1 Open)

Patient Swab

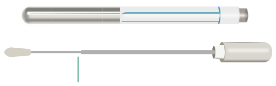

Required But Not Provided

DASH Analyzer

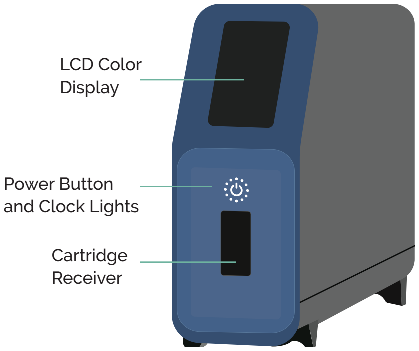

On Backside: (3) USB Connections; (1) Ethernet Port; and Power Connection

Tablet Computer

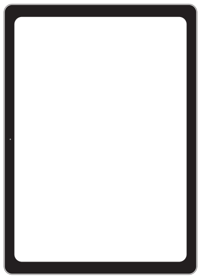

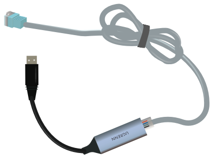

Printer

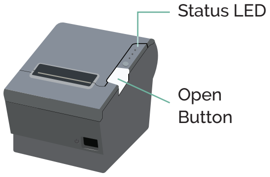

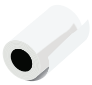

Printer Paper

Barcode Scanner

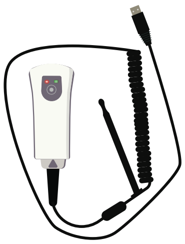

Additional items required but not provided

1. Gloves
2. Printer Paper Refills
3. DASH Power components (DASH Analyzer power adapter and power cord)
4. Ethernet components (ethernet adapter and ethernet cable)
5. Printer power adapter and power cord
6. Tablet power adapter and power cord
7. The DASH SARS-CoV-2/S Test QC Positive (REF 0314P)
8. The DASH SARS-CoV-2/S Test QC Negative (REF 0315N)

Warnings and Precautions

For *in vitro* diagnostic use.

For prescription use only.

Positive results are indicative of presence of SARS-CoV-2 RNA.

Laboratories within the United States and its territories are required to report all test results to the appropriate public health authorities.

This product has not been FDA cleared or approved; but has been authorized for emergency use by FDA under an EUA for use by authorized laboratories.

This product has been authorized only for the detection of nucleic acid from SARS-CoV-2, not for any other viruses or pathogens.

The emergency use of this product is only authorized for the duration of the declaration that circumstances exist justifying the authorization of emergency use of in vitro diagnostics for detection and/ or diagnosis of COVID-19 under Section 564(b)(1) of the Federal Food, Drug and Cosmetic Act, 21 U.S.C. § 360bbb-3 (b)(1), unless the declaration is terminated, or authorization is revoked sooner.

---

1. Before performing this test, read the DASH SARS-CoV-2/S Test Instructions for Use completely.
2. For Use Under an Emergency Use Authorization (EUA) Only.
3. Store DASH SARS-CoV-2/S Test Cartridges and DASH Analyzer at room temperature (between 15˚C and 30˚C)
4. Operate the DASH Analyzer on a level surface
5. DO NOT use a Cartridge that is wet or has leaked.
6. DO NOT use a Cartridge whose top Covers #1 and #2 are not intact.
7. DO NOT use a Cartridge that has been dropped.
8. DO NOT open Cartridge Cover #1, until you are ready to perform testing.
9. DO NOT open Cartridge Cover #2, until you are ready to insert Cartridge into the Analyzer.
10. Run cartridge immediately after Cartridge Cover #2 is removed.
11. DO NOT flip Cartridge over after peeling off Covers #1 and #2.
12. DO NOT hold the Cartridge with the back end next to the Cover #2.
13. Treat all specimens as potentially infectious. Follow universal precautions when handling samples, this kit and its contents.
14. Proper sample collection, storage and transport are essential for correct results.
15. Leave test Cartridge sealed in its foil pouch until just before use. Do not use if pouch is damaged or open.
16. Do not use kit past its expiration date.
17. Do not reuse a used test Cartridge.
18. Inadequate or inappropriate sample collection, storage, and transport may yield false test results.
19. All components of this kit should be discarded as Biohazard waste according to Federal, State and local regulatory requirements. Solutions used to make the positive control swab are non-infectious. However, patient samples, controls, and test Cartridges should be handled as though they could transmit disease. Observe established precautions against microbial hazards during use and disposal.
20. Wear appropriate personal protection equipment and gloves when running each test and handling patient specimens. Change gloves between handling of specimens suspected of COVID-19.
21. Swabs in the kit are for use with DASH SARS CoV-2/S Test. Do not use other swabs.

⚠ Keep the work area clean to prevent contamination.

⚠ Wear gloves while handling samples and change gloves between testing each specimen.

REF IVD Rx ONLY

Chemical Hazards

Signal Word: WARNING

UN GHS Hazard Statements

- Harmful if swallowed.
- May be harmful in contact with skin.
- Causes eye irritation.

UN GHS Precautionary Statements

Prevention

- Wash hands thoroughly after handling.

Response

- Call a **POISON CENTER** or doctor/physician if you feel unwell.
- If skin irritation occurs: Get medical advice/attention.
- **IF IN EYES:** Rinse cautiously with water for several minutes. Remove contact lenses, if present and easy to do. Continue rinsing.
- If eye irritation persists: Get medical advice/attention.

Sample Collection, Transport and Handling

Specimen Collection and Handling

Test specimens immediately after collection for optimal test performance. Inadequate specimen collection or improper sample handling/storage/transport may yield erroneous results. Refer to the CDC Interim Guidelines for Collecting, Handling, and Testing Clinical Specimens from Persons for Coronavirus Disease 2019 (COVID-19).

https://www.cdc. gov/coronavirus/2019-nCoV/lab/ guidelines-clinical- specimens.html.

Anterior Nasal (Nares) Swab

Only the swab provided in the kit is to be used for nasal swab collection. Insert the entire collection tip of the swab provided (usually ½ to ¾ of an inch, or 1 to 1.5 cm) inside the nostril. Firmly sample the nasal wall by rotating the swab in a circular path against the nasal wall at least 4 times. Take approximately 15 seconds to collect the specimen. Be sure to collect any nasal drainage that may be present on the swab. Repeat in the other nostril using the same swab. Place swab, tip first, into the swab container provided.

https://www.cdc.gov/coronavirus/2019-ncov/lab/ guidelines-clinical-specimens.html

Specimen Transport and Storage

For best performance, direct nasal swabs should be tested immediately after collection.

Quality Control - Internal Controls

The DASH SARS-CoV-2/S Test Cartridge contains a positive procedural armored RNA® control (non-infectious RNA encapsulated in MS2 bacteriophage) that is used to verify assay steps including viral lysis, RNA extraction, and nucleic acid amplification and detection were executed properly and without sample derived inhibition. The Procedural Control will display Valid on the results screen in a SARS-CoV-2 negative sample or SARS-CoV-2 positive sample. The Procedural Control is Valid if it meets the validated acceptance criteria.

Quality Control - External Controls

Controls must be used to show that the DASH SARS-CoV-2/S Test is working properly. The DASH SARS-CoV-2/S Test Positive Control Swab and DASH SARS-CoV-2/S Test Negative Control Swab are available separately.

The DASH SARS-CoV-2/S Test Positive Control (Ref 0314P) and Negative Control (Ref 0315N) Swabs must be stored at room temperature (between 15˚C and 30˚C). Controls are tested using the same procedure as for a patient sample.

It is recommended that external controls be tested at the frequency noted below.

- Each time a new lot or a new shipment of DASH SARS-CoV-2/S Test is received.
- Each time a new operator is performing the test.
- When the Analyzer is used for the first time.
- When the software is upgraded.
- When problems are suspected or identified.
- As deemed additionally necessary by internal quality control procedures, and in accordance with Local, State and Federal regulations or accreditation requirements.

If correct control results are not obtained, repeat the test using a new Control Swab, and a new test Cartridge. If the control testing continues to fail, do not perform additional clinical specimen tests or report results. Contact technical support at techservice@m2dx.com or 1-888-992 DASH.

The DASH System

The DASH System is built around an automated analyzer, which extracts specimens and performs nucleic acid amplified tests (NAAT) in assay Cartridges. It is connected to a barcode reader, which scans specimen and Cartridge labels, and a thermal printer that outputs reports. A tablet computer is used to configure the DASH Analyzer settings and review test results. The tablet computer can be disconnected and stored away when not in use.

The DASH Analyzer should be setup on a clean and level surface at least 4 feet wide and 2 feet deep. Three 120VAC outlets are required where power adapters for the analyzer, printer and tablet can be plugged in. Only use Minute Molecular supplied charging cable and power adapter.

The environmental conditions for operating DASH SARS-CoV-2/S Test are 15-30˚C, 20%-80% relative humidity.

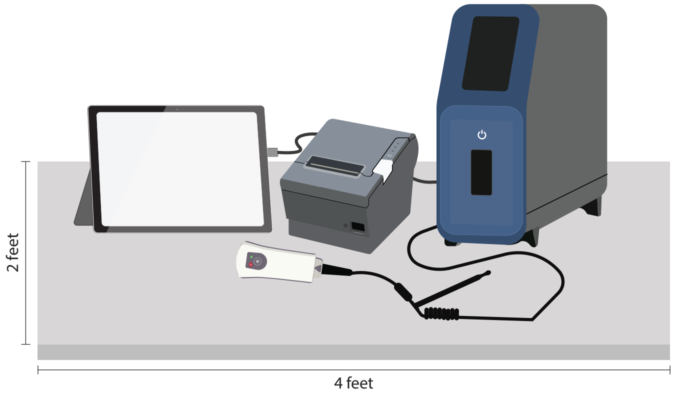

1. Unpacking Instructions

Take all components out of their shipping containers and identify them to be sure you have received everything.

To Unpack, read the steps below and watch the following **tutorial video**:

1.1 Unpack the DASH Analyzer

Take hold of the top of the DASH Analyzer and lift it out of its shipping container. Then, remove the protective wrapping.

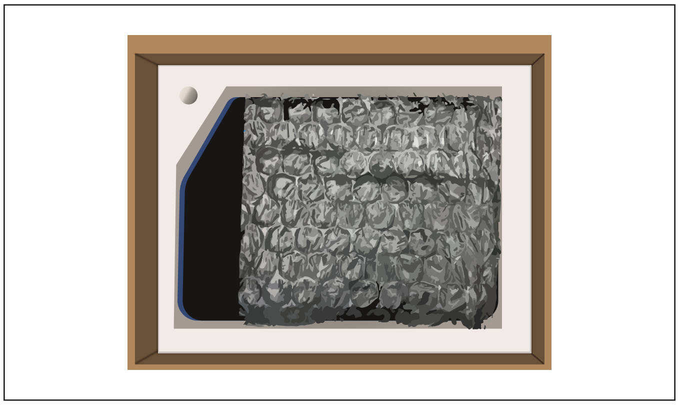

Place the DASH Analyzer in the center of a level surface at least 2 feet deep and 4 feet wide. There should be three 120VAC power outlets to plug in the DASH Analyzer, the Printer, and the Tablet.

1.2 Unpack the Accessories

The Accessories Shipping Carton contains the Epson Printer, the DASH Power components, and the Ethernet components.

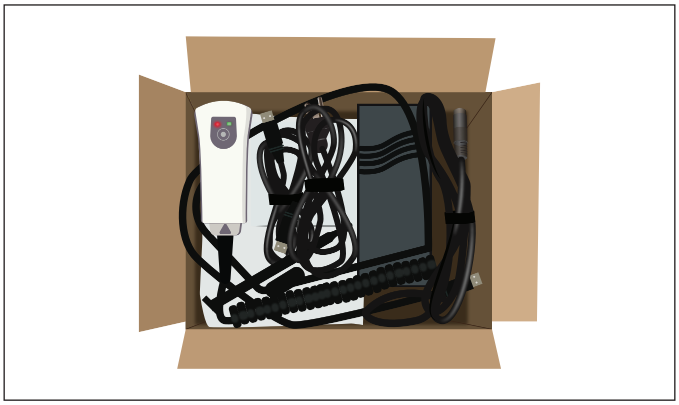

1.2.1 Unpack the DASH Analyzer power adapter and power cord

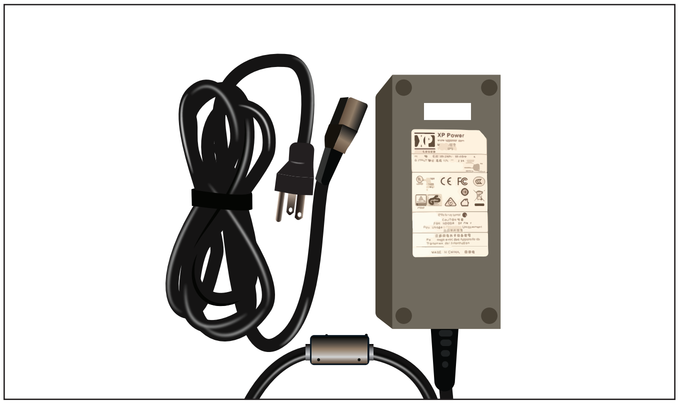

The DASH Analyzer power adapter has the XP Power logo on it.

1.2.2 Unpack the barcode scanner and USB printer cable

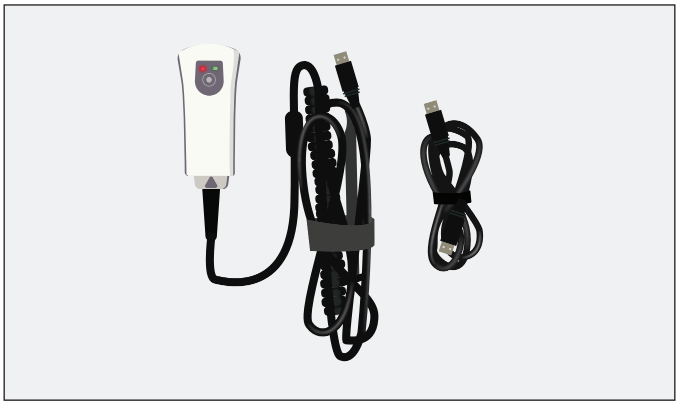

1.2.3 Unpack the Ethernet adapter and Ethernet cable

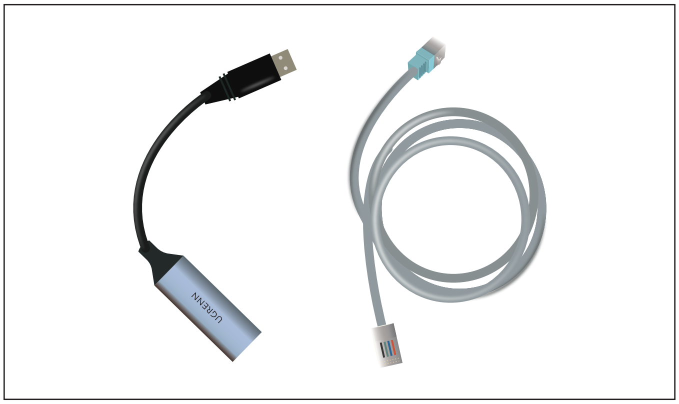

1.2.4 Unpack the Epson Printer box and unbox the printer, power adapter and power cord

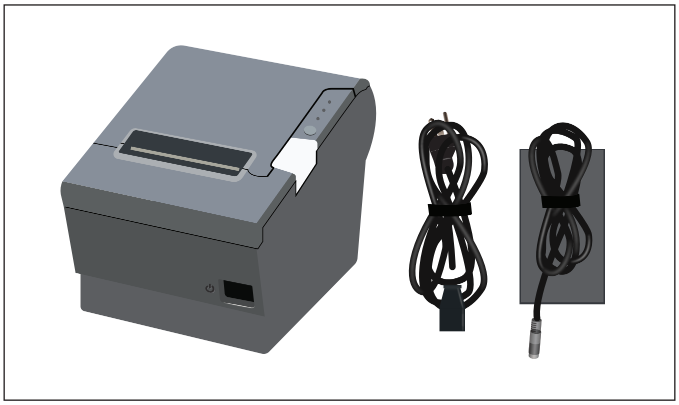

One roll of **thermal paper** is shipped in the printer.

Additional paper can be ordered in cartons of 10 rolls.

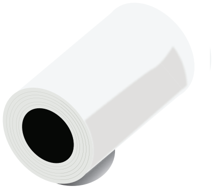

1.3 Unpack the PC Tablet

Open the Microsoft Surface Pro box and unpack the tablet, power adapter, and power cord.

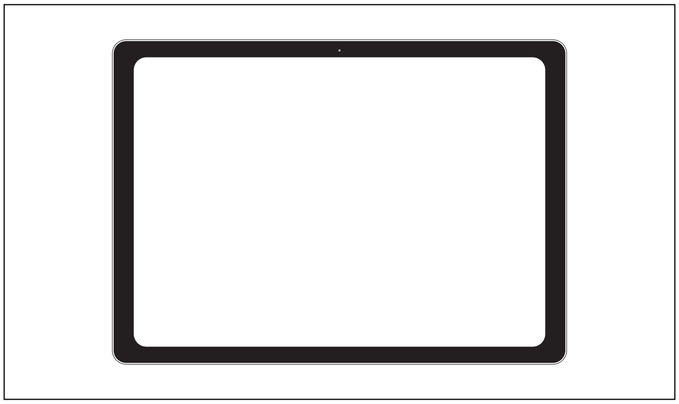

2. Installation and Set-Up

After unpacking and identifying all the components, set up the printer, barcode scanner, Ethernet connection and PC tablet.

Warning: DASH Analyzer must be located on a level surface.

Place the DASH Analyzer on the right side of your work area an angle with the back side facing to the left and forward. To the left of the DASH Analyzer, place the printer upside down. To the left of the printer, place the tablet. The power adapters should be placed behind the units. The barcode reader can be placed in the front.

The system is most easily set up by as shown in the picture below:

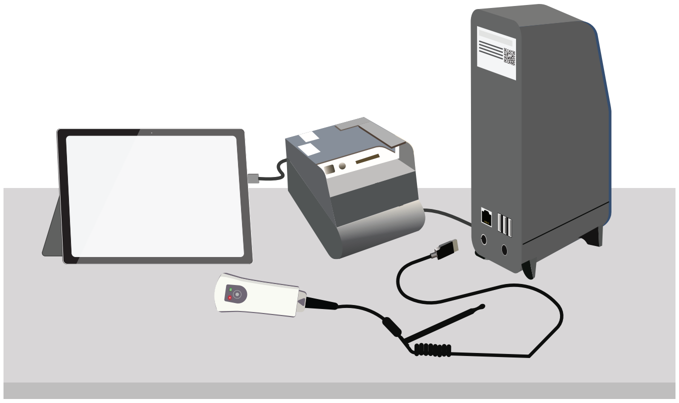

To Set Up, follow the steps below and watch the following **tutorial video**:

2.1 Set-Up the DASH Analyzer

Turn the DASH Analyzer so the back side is facing forward to make it easier to plug in cables.

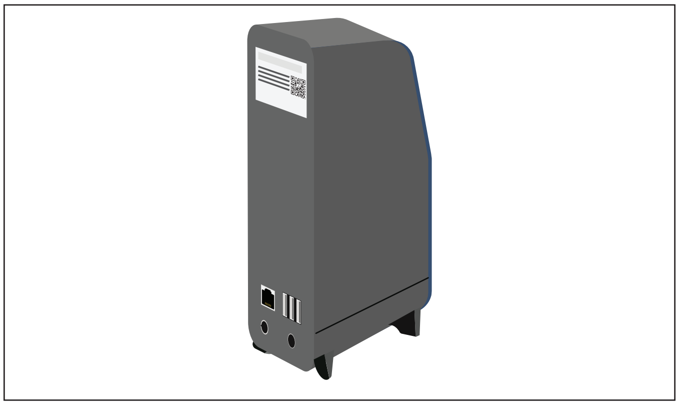

2.1.1 Connect the DASH Analyzer's power adapter to the round socket on the back of the DASH Analyzer. The flat side of the plug should be down.

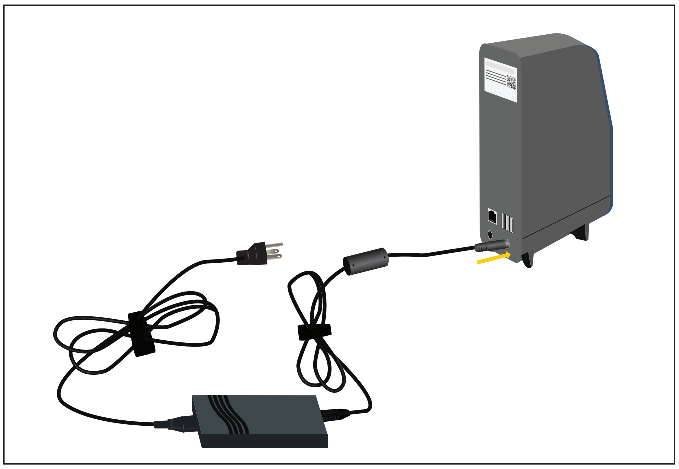

Connect the power cord to the adapter, then place it behind the DASH Analyzer.

2.1.2 Connect the barcode reader to any one of the USB ports and set it in front of the work area.

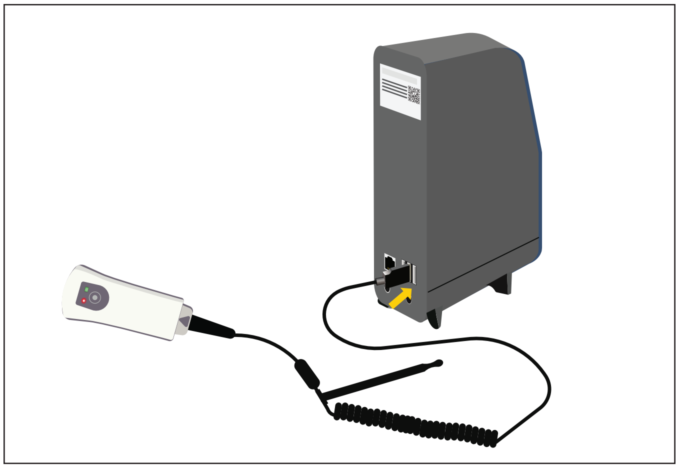

2.2 Set-Up the Printer

Turn the printer upside down so it's easier to plug in the cables.

2.2.1 Insert USB cable's square plug into the printer's square socket, with the USB connect icon facing the bottom of the printer.

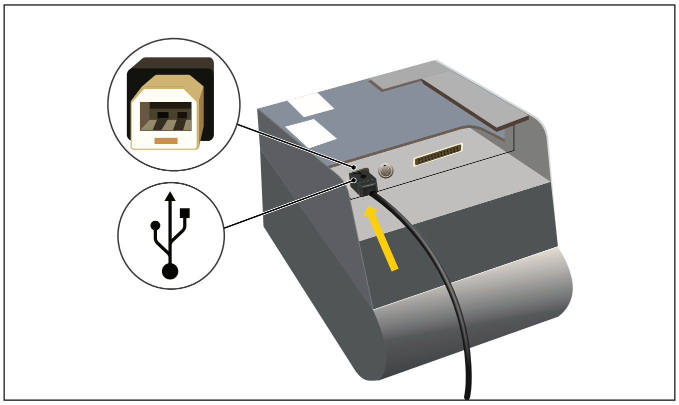

2.2.2 Plug the other side of the USB cable into the back of the DASH Analyzer.

2.2.3 Insert the round Plug of the Power Adaptor cable into the printer.

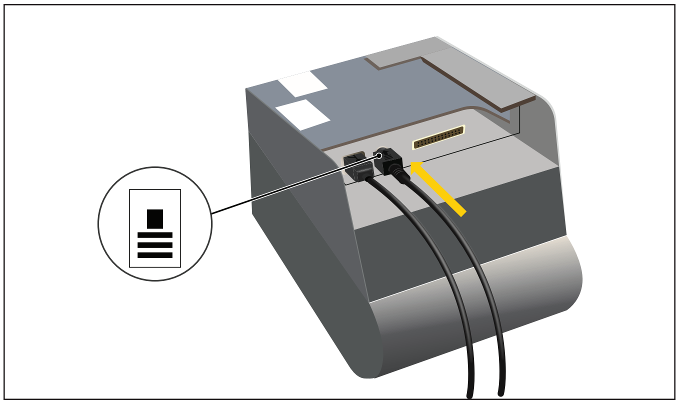

The flat side with the arrow should face the bottom of the printer

2.2.4 Plug the Power Cord into the Power Adapter.

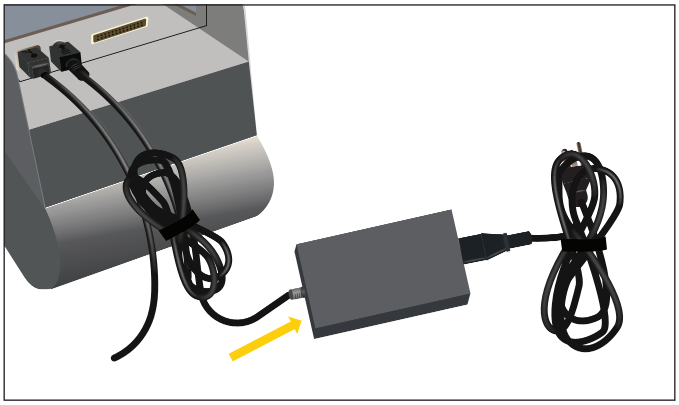

Turn the printer right side up.

2.2.5 Plug power cord into 120VAC outlet

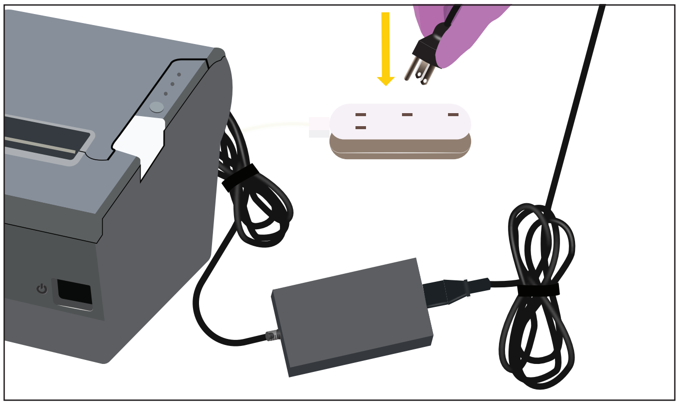

2.2.6 Power up the printer

Push the white dot side of the power switch on the front of the printer

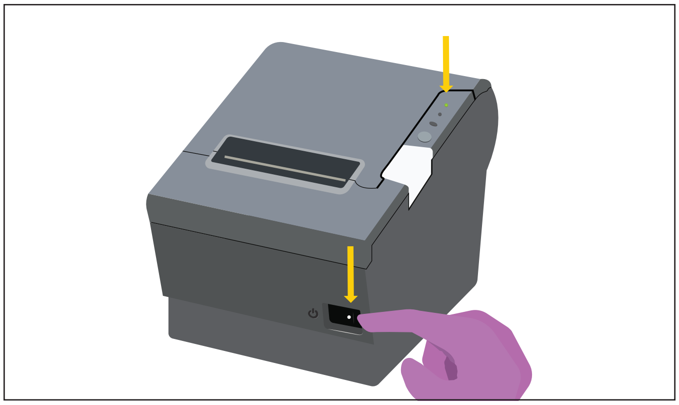

Green LED on the top right side will illuminate when power is on

2.2.7 Check thermal paper

Open the lid by pressing on the grey button on the right side of the printer

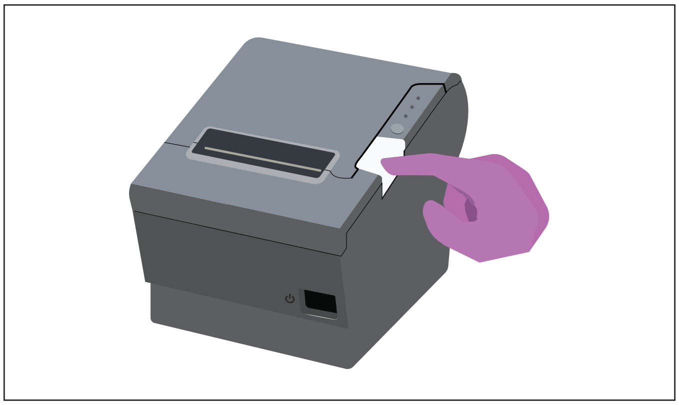

Note that the end of the roll of thermal paper is feeding from the bottom towards the front. Pull the end to unroll 6 inches paper.

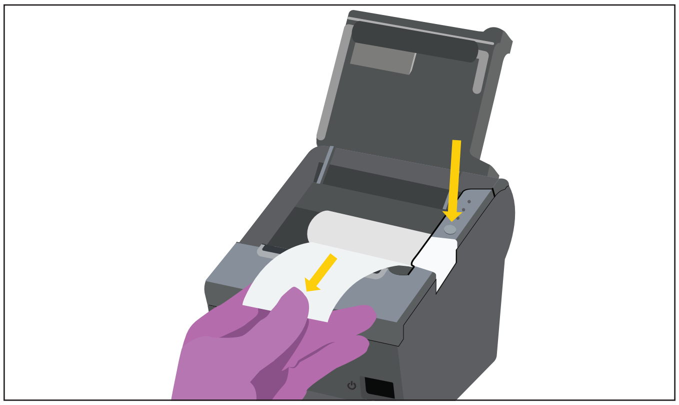

Close the Printer lid.

Tear off excess paper by pulling it forward over the tear bar and slightly to the side.

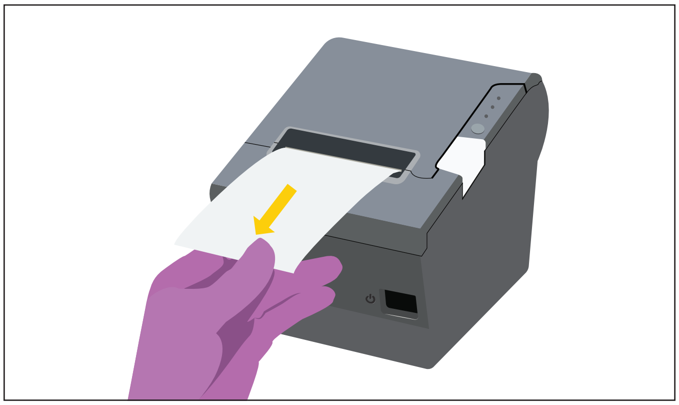

2.3 Set-Up the PC Tablet

The PC Tablet should be placed on the left side of DASH Analyzer where it is connected with the DASH Analyzer through the Ethernet cable. It is also connected to a power adapter that is plugged into 120VAC.

2.3.1 Place the tablet on the left side of DASH Analyzer and pull out the kickstand to have the screen at a 45˚ angle. Adjust the angle to minimize glare and facilitate typing on the screen.

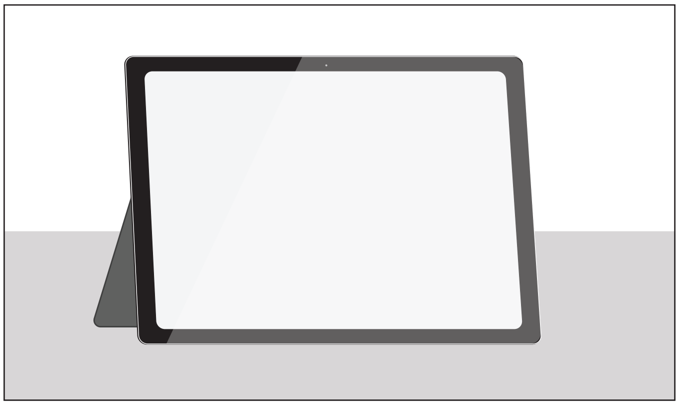

2.3.2 Plug one end of the Ethernet cable into the USB-Ethernet adapter and the other end into the socket on the left side of the back panel of the DASH Analyzer

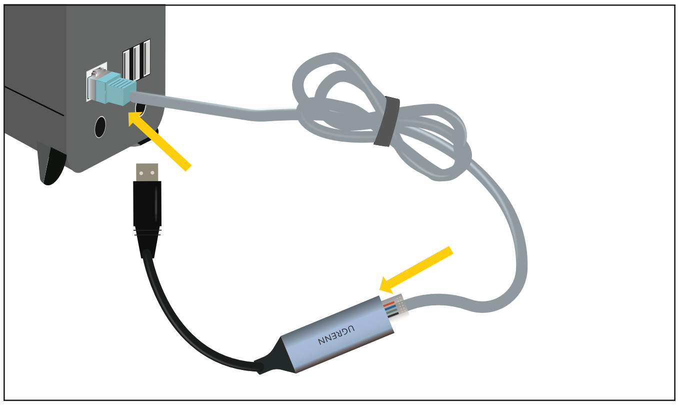

2.3.3 Plug the other end of the USB-Ethernet Adapter to the USB port in the side of the tablet.

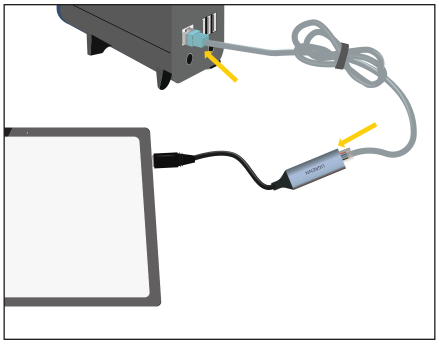

2.3.4 Connect the power adaptor cord to the socket on the side of the tablet. Connect the Power Cord to the Power Adaptor and plug it into 120VAC.

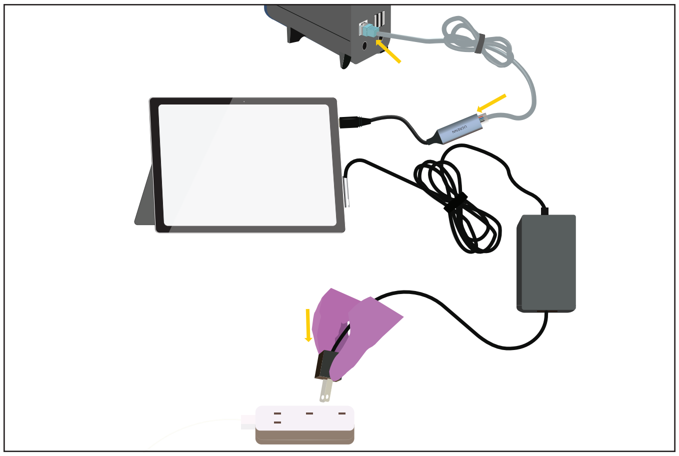

2.4 Arrange system components

- Turn the DASH Analyzer around so the display is facing the operator.
- The Printer and Barcode scanner should be to the left of the DASH Analyzer.
- The power adapters should be placed behind the printer and DASH Analyzer.
- Plug the DASH Analyzer Power Cord into a grounded 120VAC outlet.
- Fold up excess lengths of all cables and secure them with cable ties.

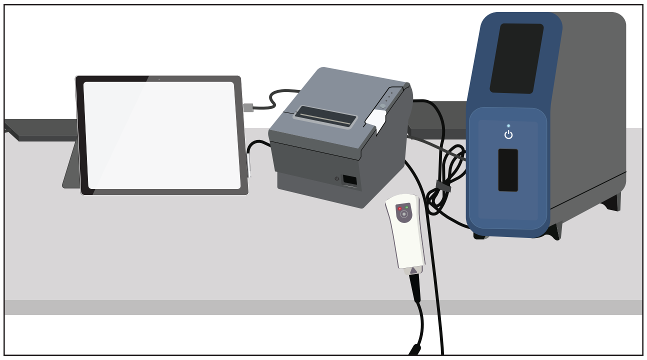

2.5 Start-Up the DASH Analyzer

To Start Up, follow the steps below and watch the following **tutorial video**:

2.5.1 Power Up

Press the power button in the center of the DASH Analyzer's Clock Lights.

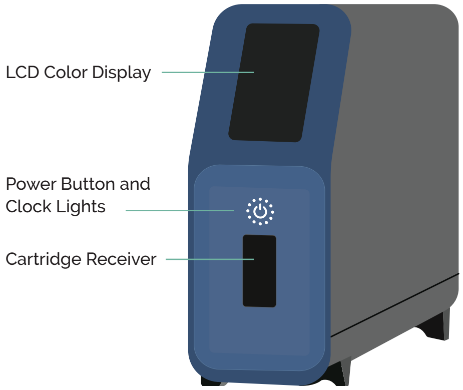

On Backside: (3) USB Connections; (1) Ethernet Port; and Power Connection

The operating system will boot up, displaying a series of screens.

2.5.2 Eject shipping Cartridge

After initialization is completed in approximately 55 seconds, the DASH Analyzer will display an "Eject Cartridge" button. Press it, and door will slide open, and the Cartridge will be ejected. Remove the shipping Cartridge from the carrier and place it in the shipping container.

Note: The shipping Cartridge should be reinstalled whenever the DASH Analyzer is shipped. To reinstall the shipping Cartridge, scan the barcode of the shipping Cartridge, touch Open on the DASH Analyzer, and insert shipping Cartridge.

2.5.3 Warmup

After the shipping Cartridge is removed, the DASH Analyzer will begin warming up by turning on its heaters. When they reach their preset temperatures in approximately 80 seconds, operators can login and begin processing Cartridges.

2.5.4 Start-Up the Tablet

Users with Admin privileges can make changes to some of the DASH Analyzer's settings, including assigning users and user pins assigned to the DASH Analyzer, through the Tablet app.

2.5.5 Turn on the Tablet's power

After starting up the DASH Analyzer, the Tablet can be started up by pressing the button on the top left edge of the frame.

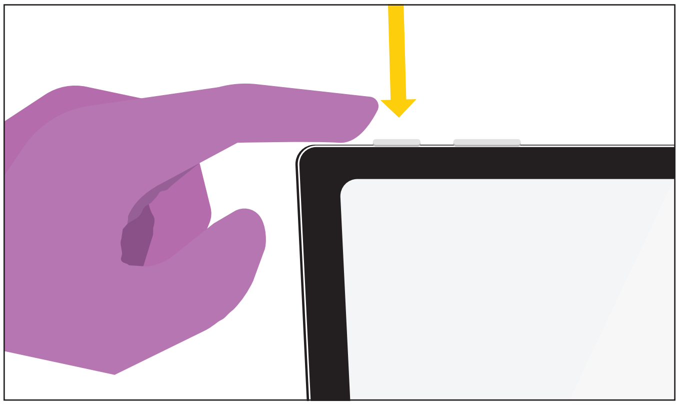

The Windows 10 operating system will begin booting up and display its lock screen. To enter the Windows password, swipe up on the lock screen.

The Windows user has been preassigned a name relevant to the system's owner, which you'll see highlighted in the bottom left corner.

The password has also been preassigned to the system's owner.

Enter the appropriate password and the Windows home screen will be displayed.

The Tablet has been preset to be in the Tablet Mode, so actions are initiated with a single touch instead of a double click, which is the more familiar Desktop Mode.

2.5.6 Open the DASH Connect App

Start the app by pressing its icon.

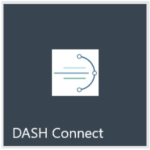

When the welcome screen shows, press the Open button.

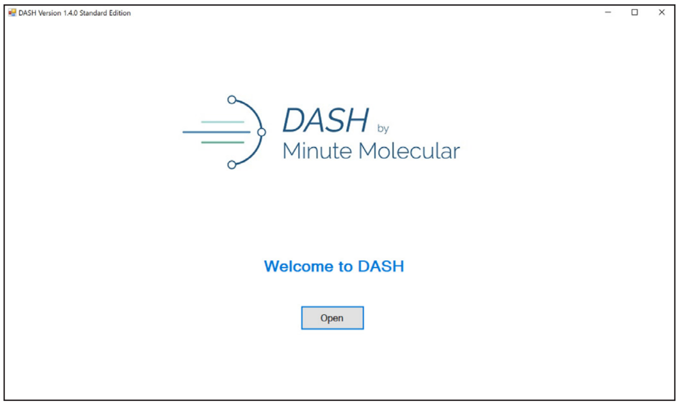

2.5.7 Connect the DASH Connect App to the Analyzer

The DASH Connect App connects to DASH Analyzer and asks for its serial number, which is then displayed in the Connect window.

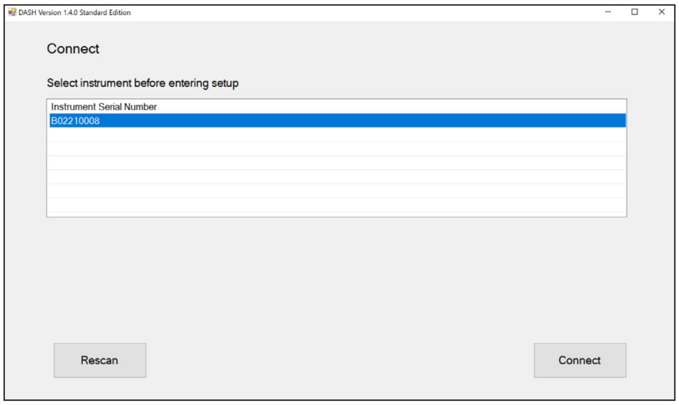

Touch the line with the serial number for the DASH Analyzer, which highlights it in blue.

Press the Connect button, which downloads user, site, test results and other information from the DASH Analyzer

2.5.8 Add a User

After information is downloaded from the DASH Analyzer, the Home screen is displayed. From here, users can be added by pressing the Users button. After clicking on the Users button, click on the Add User button to add a new user to the DASH Analyzer.

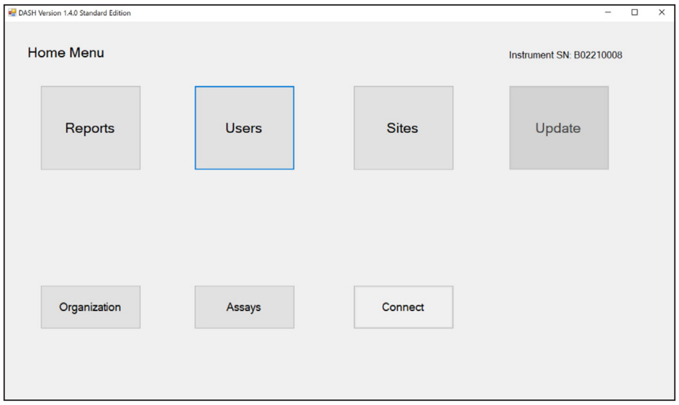

2.5.9 Assign a UserID and PIN

The Add User screen is shown below. It allows the user to add a new user(s).

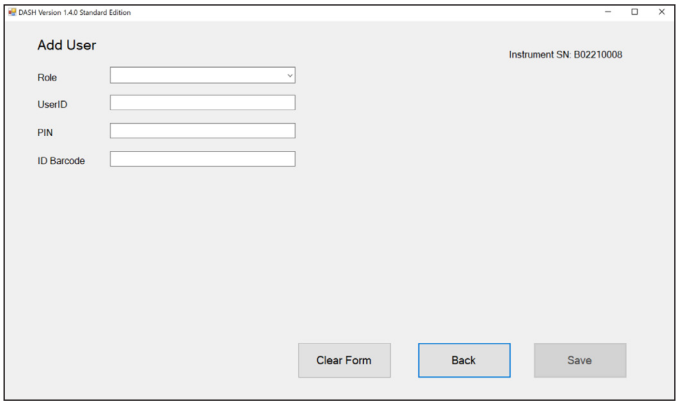

To add a user:

- Role: Select a role, 'Admin' or 'User', for the new user of the DASH Analyzer
- UserID: Enter a User ID that will be displayed on the DASH Analyzer.
- PIN: Assign a 4-digit, numeric code. The user will have to select their UserID and enter their 4-digit PIN when running a test on this DASH Analyzer. Please make notes of the UserIDs and PINs for later use on the DASH Analyzer. After Role, UserID, and PIN have been filled, the "Save" button will turn green.
- ID Barcode: This field is optional. If User will have a scannable barcode ID, then type the ID in this field.

2.5.10 Save your UserID and PIN

After clicking the "Save" button, a pop-up window will appear to notify the user that to update the Analyzer with changes go to the Home Menu screen.

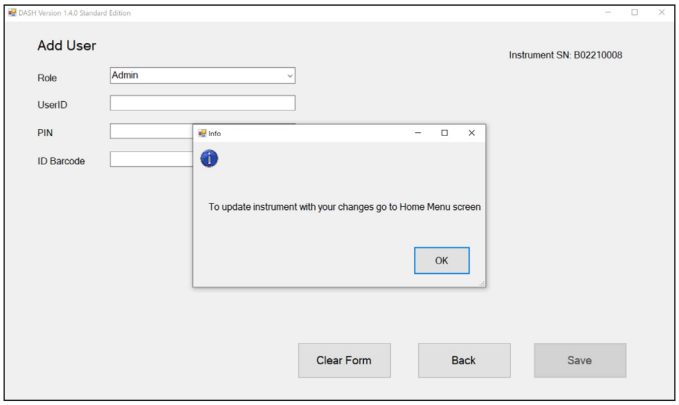

2.5.11 Transfer the new UserID and PIN to the DASH Analyzer

Click the "Home" button to go to the Home screen, the "Update" button will appear in green.

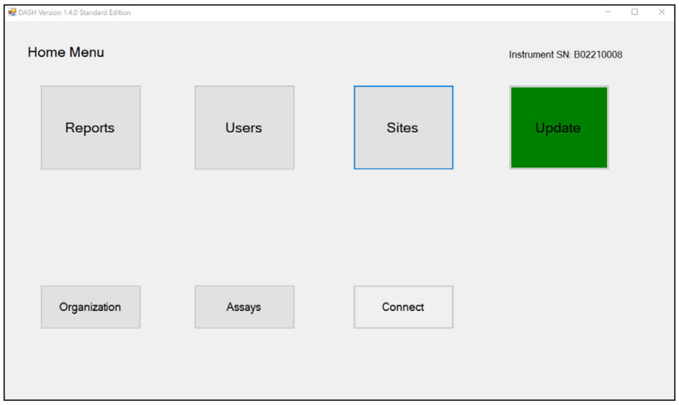

2.5.12 To exit the DASH Connect App, click the close tab on the upper right corner, and a pop-up window will appear to confirm.

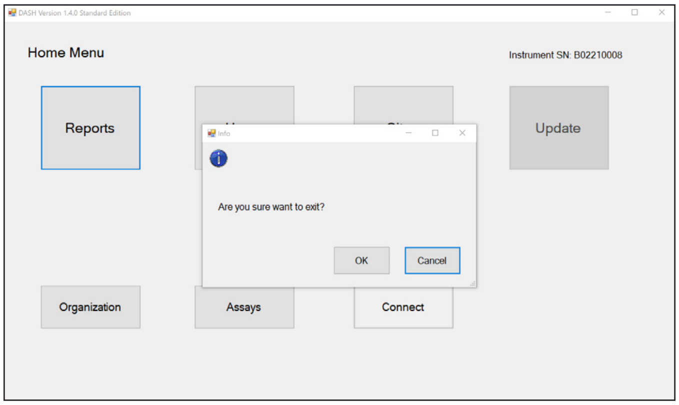

For further instructions on how to edit or delete Users or other settings and reports available in the App, see Section 6.

3. Running QC Samples

To see how to run a QC test, follow the steps below and watch the following **tutorial video**:

3.1 Put on gloves (PPE) and gather materials needed to run a QC Test.

Identify the boxes (example below) labeled as SARS-CoV-2/S QC Negative control (REF 0315N) and SARS-CoV-2/S QC Positive Control (REF 0314P).

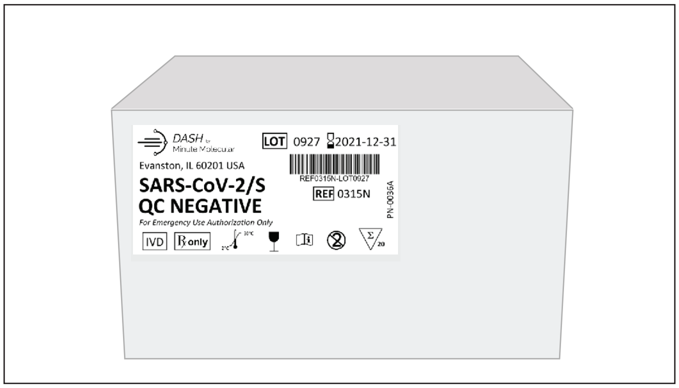

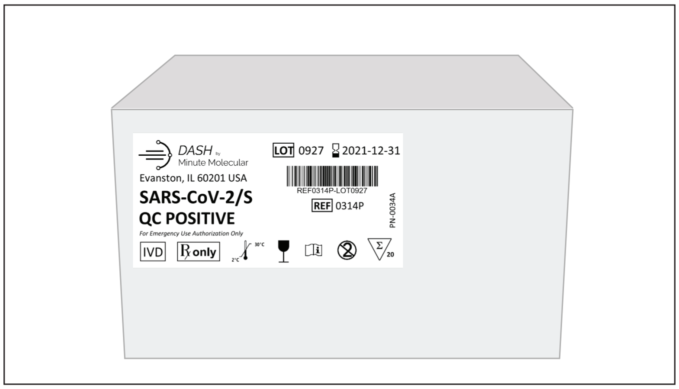

Pick either a SARS-CoV-2/S QC Negative (top) control or a SARS-CoV-2/S QC Positive (bottom) control, as pictured below, from the respective box. Note these Negative and Positive controls are in white packaging.

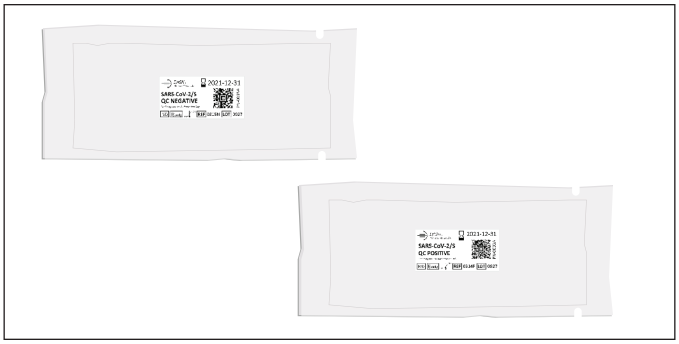

Next, identify the Cartridge boxes (REF 0121S) labeled as SARS-CoV-2/S (pictured below).

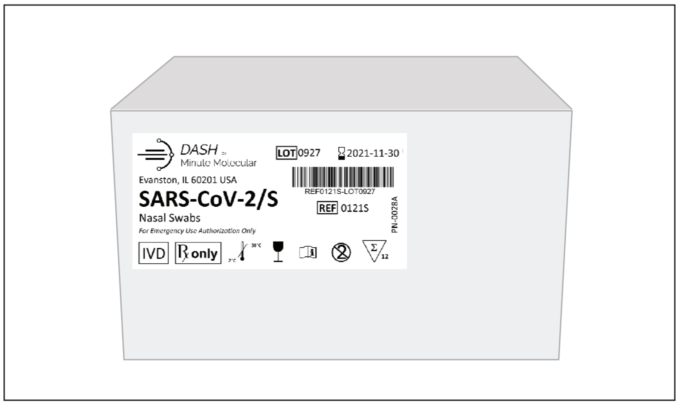

Pick a Cartridge package, as pictured below, from the Cartridge box. Note the Cartridge is in silver packaging.

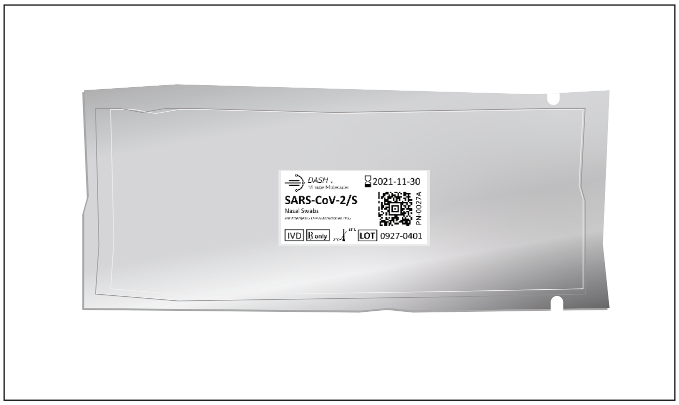

3.2 When the DASH Analyzer is switched on, or after another User has logged out, the drop down list of username will be displayed. Select the username, and enter the 4-digit PIN and touch "Enter". If assigning IDs and Pins for the first time, see 2.5.8.

Your username is now shown in the header. Available tests are shown in the main section. The Analyzer will then warm up.

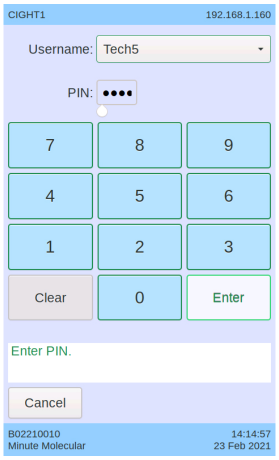

3.3 Scan the SARS-CoV-2/S QC Negative Control or a SARS-CoV-2/S QC Positive Control QC swab packaging by holding down the button on the barcode reader. Note the bar code reader should be about 5-6 inches from the item being scanned.

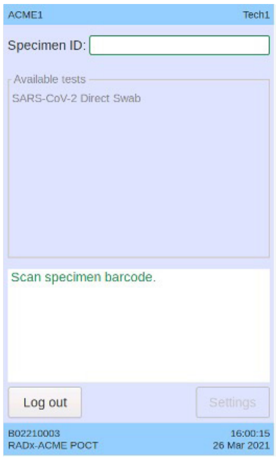

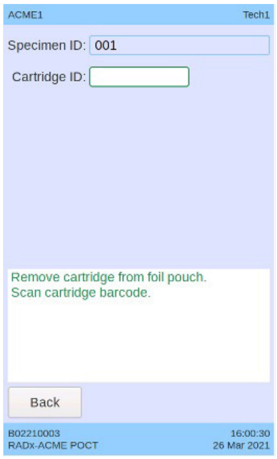

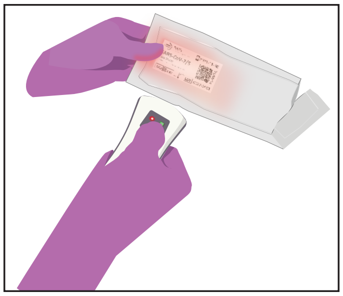

After scanning the QC control, the specimen ID is shown in the window.

3.4 Remove Cartridge from the silver packaging

Next, remove the Cartridge from the silver packaging and scan the Cartridge Barcode. Note the bar code reader should be about 5-6 inches from the item being scanned.

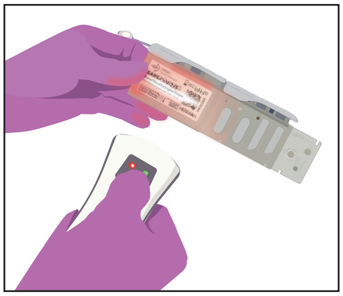

Once cartridge is removed from the silver packaging it should be run immediately.

Please note the correct way to handle the Cartridge

How to handle the Cartridge

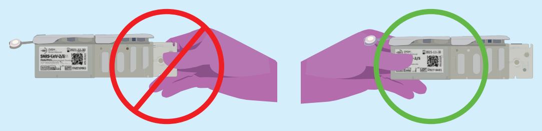

3.5 Prepare the SARS-CoV-2/S QC Negative control or a SARS-CoV-2/S QC Positive Control QC Swab for USE in the cartridge.

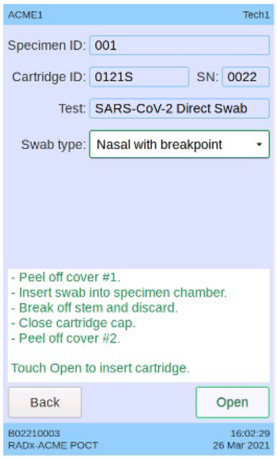

Follow the instruction on the screen of the DASH Analyzer to prepare the specimen QC control swab for the Cartridge.

Please note the key areas of the Cartridge in the illustration below.

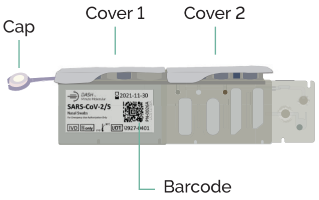

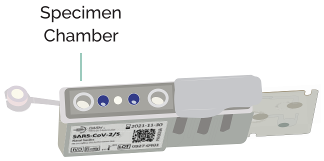

(Top View of Cartridge With Cover 1 Open)

3.5.1 First, Place Cartridge on flat surface and peel off Cover #1. DO NOT flip Cartridge over after peeling off Cover #1, as this part of the Cartridge has liquid in it.

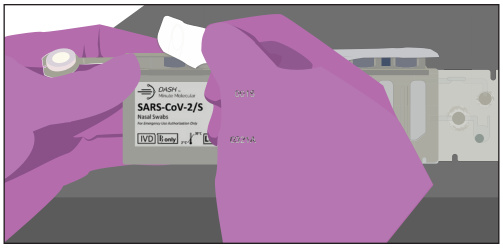

3.5.2 Next, open the white packaging for the SARS-CoV-2/S QC Negative control or a SARS-CoV-2/S QC Positive Control and remove swab from packaging.

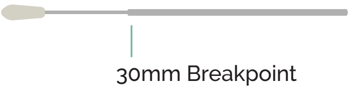

Insert QC Swab into specimen chamber, which is the opening closest to the cap.

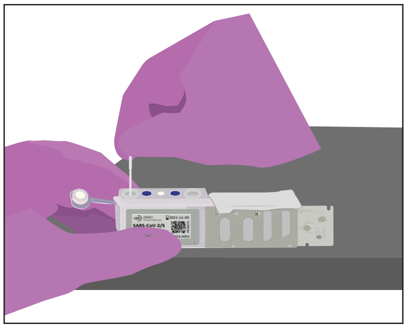

3.5.3 Next, break off the stem at the 30 mm breakpoint by using the rim of the specimen chamber as leverage while gently bending and then twisting the stem, until the stem break off and the swab tip remains in the Cartridge. Discard the stem in biohazard waste.

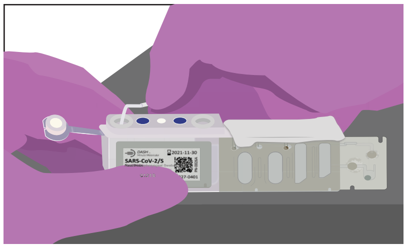

3.5.4 Close Cartridge Cap

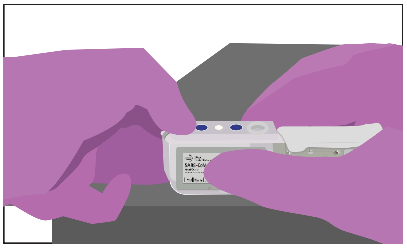

3.5.5 Peel off Cover #2. **Once Cover#2 is removed, the test should be run immediately.**

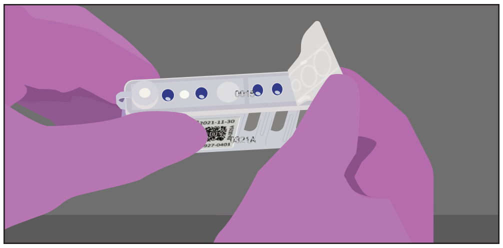

3.6 Touch "Open" to insert Cartridge

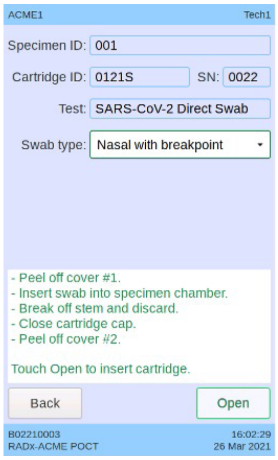

3.7 Touch "Seal Removed" button on DASH Analyzer screen

After pressing the "Open" button, "Seal removed" button displayed on screen to remind the operator to remove the Cover #2.

Touch "Seal removed" to confirm.

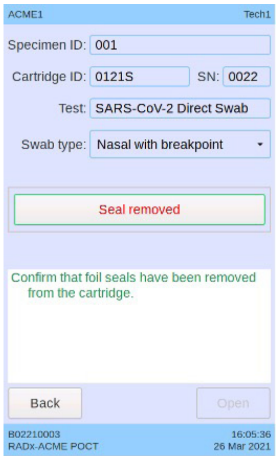

3.8 Insert the Cartridge

Door opens, the Cartridge receiver extends to wait for the Cartridge. Clock starts to count down in 24 seconds for the operator to insert the Cartridge. Insert the Cartridge.
The Cartridge receiver withdraws and the door closes.

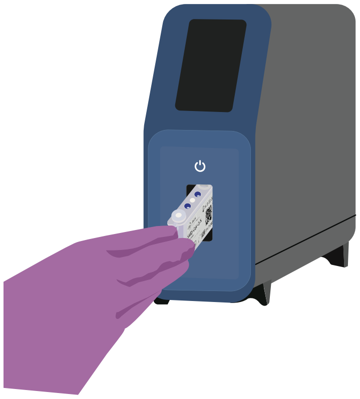

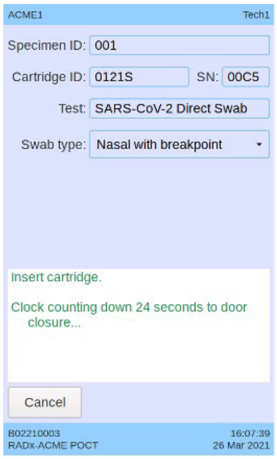

If the Cartridge receiver closes before a Cartridge has been inserted, open the Cartridge receiver again by touching "Open".

3.9 QC Test begins to process

The display shows the start time, and it starts to process. The Analyzer clock shows the progress, counting up from 1 light to 12 lights when it's done.

3.10 QC Test Result is Displayed

When the Analyzer is done analyzing, the result will be printed and displayed on the Dash Analyzer screen.

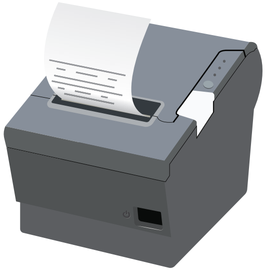

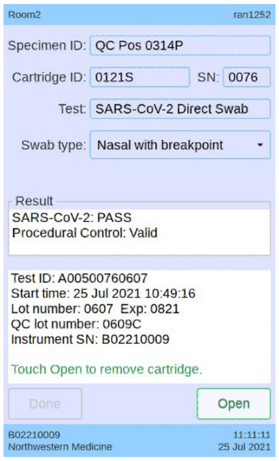

At the end of the test, the result is displayed on the screen; the results will also print automatically. The QC result should indicate SARS-CoV-2 "Pass" and Procedural Control "Valid". If the QC result indicates SARS-CoV-2 "Fail" or Procedural Control "Invalid", user should change gloves and repeat. If the QC results fails a second time, contact technical support at techservice@m2dx.com or 1-888-992 DASH.

Touch "Open" to remove the Cartridge.

3.11 Remove the Cartridge

After pressing "Open", the Cartridge receiver extends. Take out the Cartridge and discard immediately in biohazard waste.

4. Running Patient Samples

To see how to run a Test in Patient Sample, follow the steps below and watch the following **tutorial video**:

4.1 Put on gloves (PPE) and gather materials needed to run a patient sample (Cartridge, swab)

Identify the Cartridge boxes labeled as SARS-CoV-2/S (REF 0121S) (pictured below).

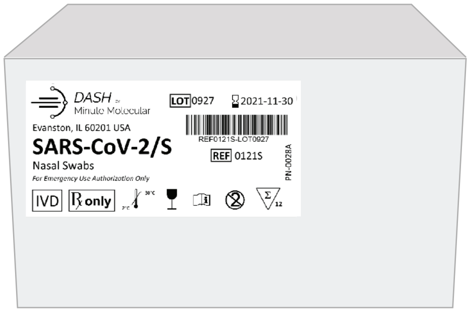

Pick a Cartridge package, as pictured below, from the Cartridge box. Note these Cartridge packaging is in silver packaging.

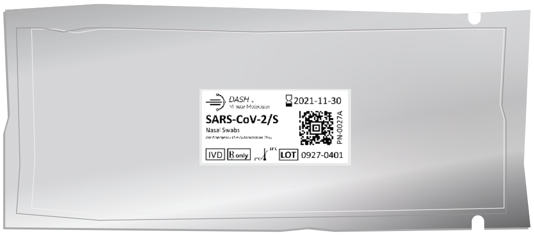

Identify the Swab box, as pictured below.

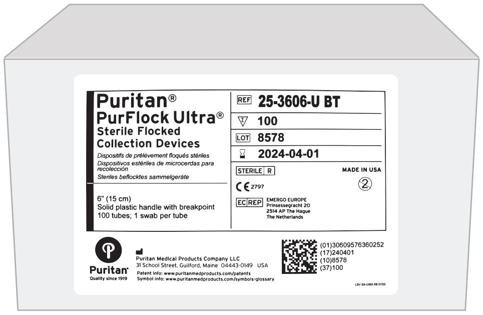

Swabs have two parts, a Swab Container and Swab with a 30 mm Breakpoint, as pictured below.

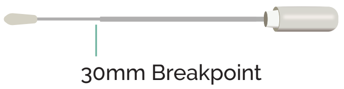

NOTE: Swabs in the kit are for use with DASH SARS CoV-2/S Test. Do not use other swabs.

4.2 Collect anterior nares sample

Insert the entire collection tip of the swab provided (usually ½ to ¾ of an inch, or 1 to 1.5 cm) inside the nostril. Firmly sample the nasal wall by rotating the swab in a circular path against the nasal wall at least 4 times. Take approximately 15 seconds to collect the specimen. Be sure to collect any nasal drainage that may be present on the swab. Repeat in the other nostril using the same swab. Place swab, tip first, into the swab container provided.

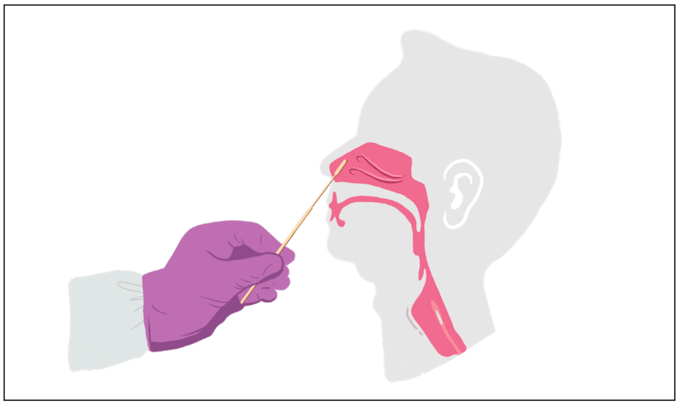

4.3 Run a DASH SARS-CoV-2/S Test

When the DASH Analyzer is switched on, or after another User has logged out, the drop down list of username will be displayed. Select the username and enter the 4-digit PIN and touch “Enter”. If assigning IDs and pins for the first time, see 2.5.8.

Your username is now shown in the header. Available tests are shown in the main section. The Analyzer will then warm up.

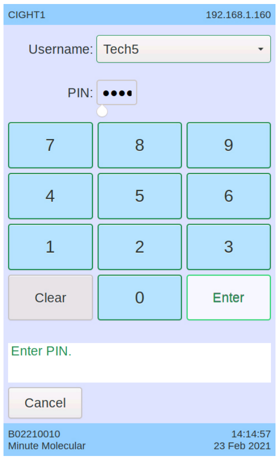

4.4 Scan the specimen barcode

Place your internal barcode labels on the specimen container.

Then, scan the specimen barcode. Note the bar code reader should be about 5-6 inches from the item being scanned. After scanning the specimen barcode, the specimen ID is shown in the window.

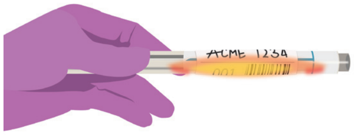

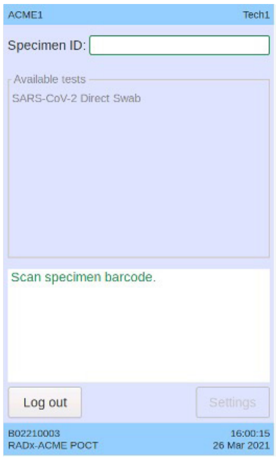

4.5 Scan the Cartridge Barcode

Next, remove the Cartridge from the foil pouch and scan the Cartridge Barcode. Note the bar code reader should be about 5-6 inches from the item being scanned. Once cartridge is removed from the silver packaging, run test immediately.

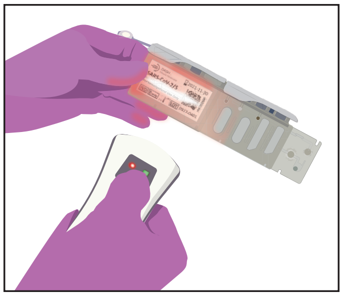

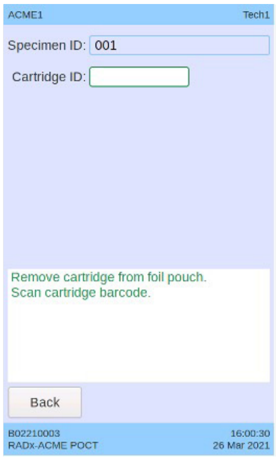

Please note the correct way to handle the Cartridge

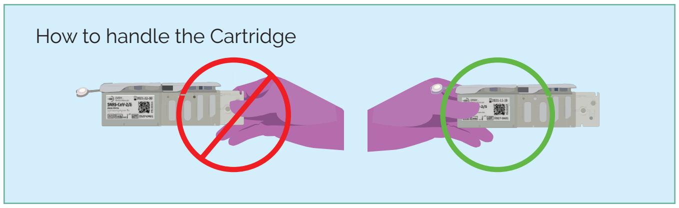

4.6 Prepare the specimen in the Cartridge

Follow the instruction to prepare the specimen in the Cartridge

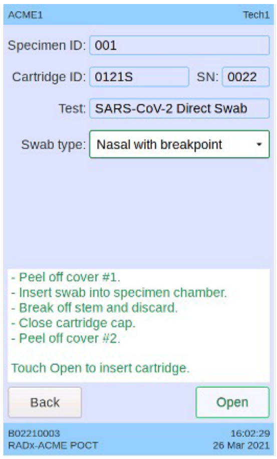

Please note the key areas of the Cartridge in the illustration below.

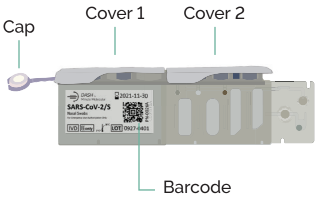

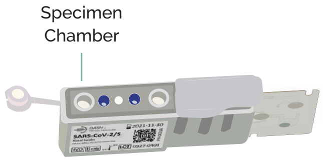

(Top View of Cartridge With Cover 1 Open)

4.6.1 Place Cartridge on flat surface and peel off Cover #1. DO NOT flip Cartridge over after peeling off Cover #1, as this part of the Cartridge has liquid in it.

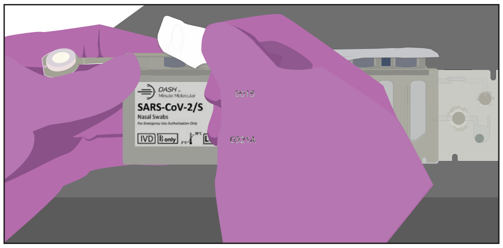

4.6.2 Insert Swab into specimen chamber, which is the opening closest to the cap.

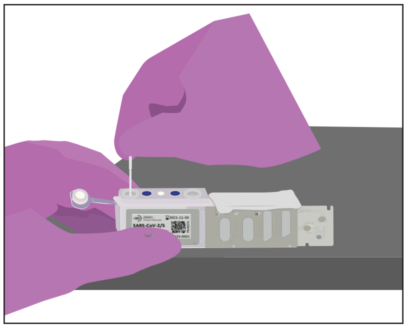

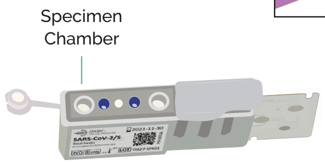

(Top View of Cartridge With Cover 1 Open)

4.6.3 Next, break off the stem at the 30 mm breakpoint by using the rim of the specimen chamber as leverage while gently bending and then twisting the stem, until the stem break off and the swab tip remains in the Cartridge. Discard the stem.

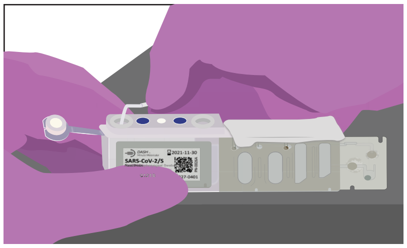

4.6.4 Close Cartridge Cap

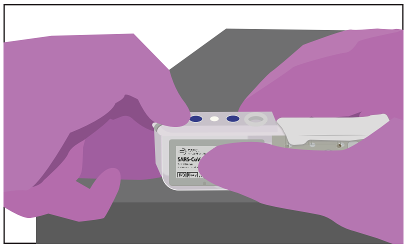

4.6.5 Peel off Cover #2. **Once Cover#2 is removed, the test should be run immediately.**

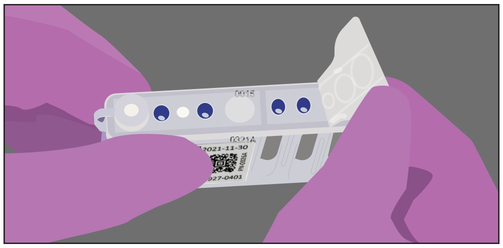

4.7 Touch "Open" to insert Cartridge

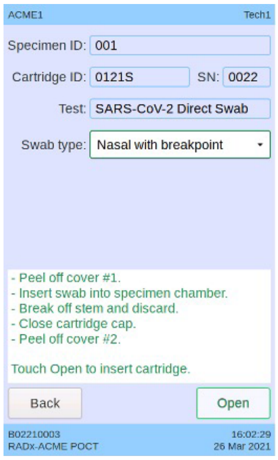

4.8 Touch “Seal Removed” button on DASH Analyzer screen

After pressing the “Open” button, “Seal removed” button displayed on screen to remind the operator to remove the Cover #2.

Touch “Seal removed” to confirm.

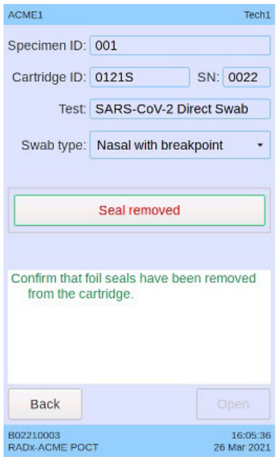

4.9 Insert the Cartridge

When the door opens, the Cartridge receiver extends out to wait for the operator to insert the Cartridge. The clock starts to count down 24 seconds for the operator to insert the Cartridge.

The Cartridge receiver withdraws and the door closes.

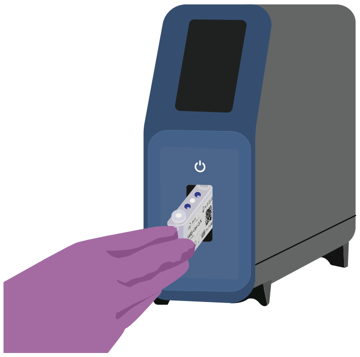

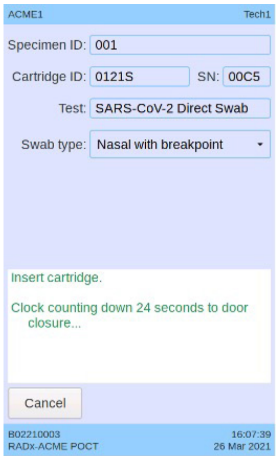

4.10 Test begins to process

The display shows the start time, and it starts to process. The Analyzer clock shows the progress, counting up from 1 light to 12 lights when it’s done.

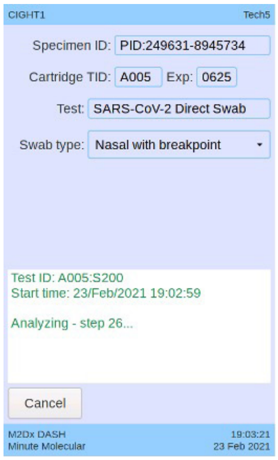

4.11 Test result displayed

At the end of the test, the result is displayed on the screen; the results will also print automatically. The specimen is POSITIVE (Or NEGATIVE Or Invalid Or Error) for SARS-CoV-2.

Touch “Open” to remove the Cartridge

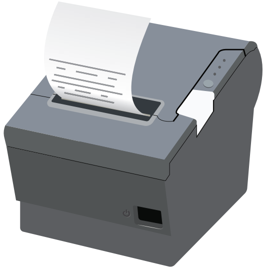

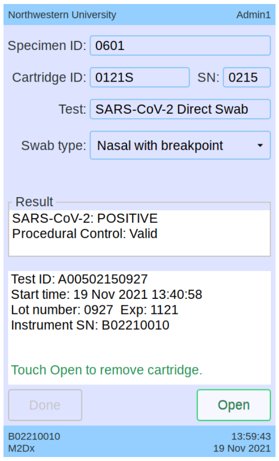

4.12 Remove the Cartridge

After pressing “Open”, the Cartridge receiver extends. Take out the Cartridge and discard immediately in biohazard waste.

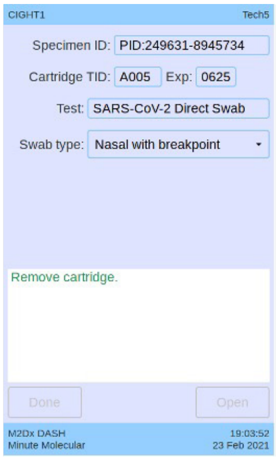

Result Interpretation

SARS-CoV-2 Positive

The 2019 novel coronavirus (SARS-CoV-2) target nucleic acids are detected. Report results to healthcare providers and relevant public health authorities.

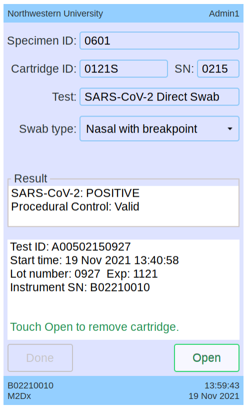

SARS-CoV-2 Negative

The 2019 novel coronavirus (SARS-CoV-2) target nucleic acids are not detected. Report results to healthcare providers and relevant public health authorities.

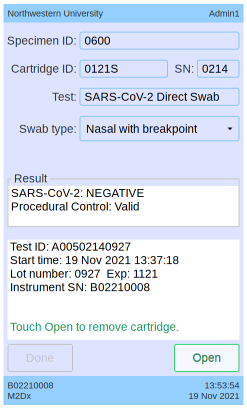

Invalid

Presence or absence of the 2019 novel coronavirus (SARS-CoV-2) target nucleic acids cannot be determined. Repeat test with a new nasal specimen and new Cartridge. Report results to healthcare providers and relevant public health authorities.

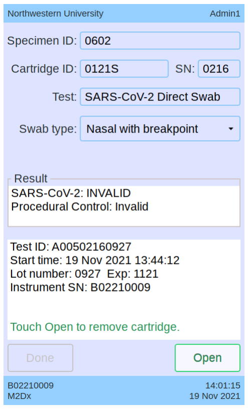

NOTE: The test results will be displayed on the DASH Analyzer touchscreen and printed after the test has been completed. The Procedural Control will display Valid on the results screen in a SARS-CoV-2 negative sample or SARS-CoV-2 positive sample. The Procedural Control is Valid if it meets the validated acceptance criteria. Screenshots of potential results screens are shown above, and the possible test results are summarized in the Table of Test Results and Interpretations below.

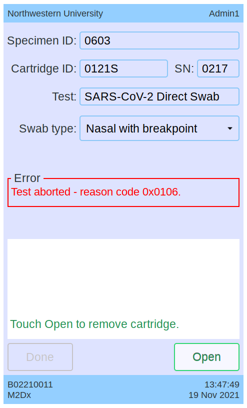

Error

Presence or absence of the 2019 novel coronavirus (SARS-CoV-2) target nucleic acids cannot be determined. Repeat test with a new nasal specimen and new Cartridge. Report results to healthcare providers and relevant public health authorities.

* If repeat test also results in an error, contact technical support at techservice@m2dx.com or 1-888-992-DASH.

RESULT	INTERPRETATION
**SARS-CoV-2 NEGATIVE**	The 2019 novel coronavirus (SARS-CoV-2) target nucleic acids are not detected.
**SARS-CoV-2 POSITIVE**	The 2019 novel coronavirus (SARS-CoV-2) target nucleic acids are detected.
**INVALID**	Presence or absence of the 2019 novel coronavirus (SARS-CoV-2) target nucleic acids cannot be determined. Repeat test with a new nasal specimen and new Cartridge.
**ERROR**	Presence or absence of the 2019 novel coronavirus (SARS-CoV-2) target nucleic acids cannot be determined. Repeat test with a new nasal specimen and new Cartridge. Analyzer detected an error and aborted the current test.

Retests

Reasons to Repeat the Test

- If any of the test results occur, repeat the test once according to instructions
- An ERROR result could be due to a system component failure.
- If an External Control fails to perform as expected, repeat external control test and/or contact technical support for assistance.

Retest Procedure

- Put on a clean pair of gloves.
- Obtain a new DASH SARS CoV-2/S Test Cartridge and process new test or control

5. Logging Out and Shutting Down

When a user is done running tests, they should sign out by touching the Log Out button. They will no longer be the active user and the button will change to Log In. DASH Analyzer will remain warmed up in a standby state for 2 hours. After that, if no one has logged in, the DASH Analyzer will go into a low power standby state.

If an operator knows that no more tests will be run for the day, they should shut down the DASH Analyzer. This is done by pressing the Power button, which then displays the Shut Down button. Touching it causes all of the DASH Analyzer internal power to be turned off.

DASH Analyzer should only be unplugged after it has been shut down and all power turned off.

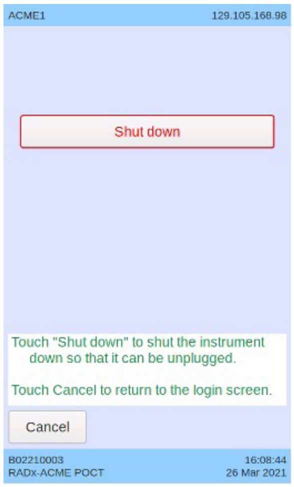

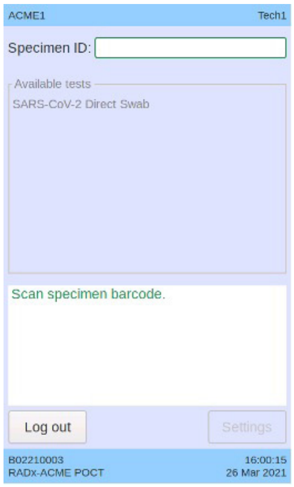

6. Editing Settings and Viewing Reports

Users with Admin privileges can make changes to some of DASH's settings through the Tablet via DASH Connect App. The DASH Connect App can also be used to view results. To access the Admin privileges, Users must log into the Tablet and the DASH Analyzer must be turned on. The Admin should not be logged in to the DASH Analyzer.

6.1 Start-Up the Tablet

6.1.1 Turn on the Tablet's power

After starting up the DASH Analyzer, the Tablet can be started up by pressing the button on the top left edge of the frame.

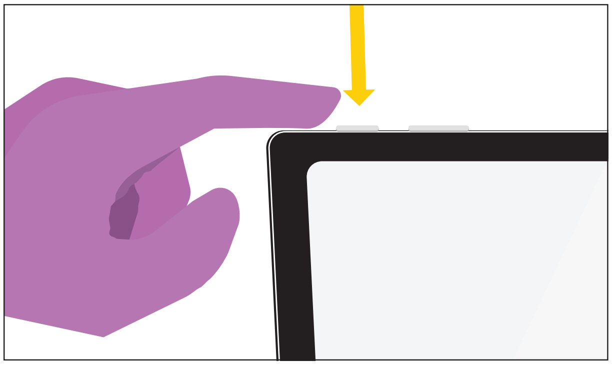

The Windows 10 operating system will begin booting up and display its lock screen. To enter the Windows password, swipe up on the lock screen.

The Windows user has been preassigned a name relevant to the system's owner, which you'll see highlighted in the bottom left corner.

The password has also been preassigned to the system's owner.

Enter the appropriate password and the Windows home screen will be displayed.

The Tablet has been preset to be in the Tablet Mode, so actions are initiated with a single touch instead of a double click, which is the more familiar Desktop Mode.

6.1.2 Open the App

Start the app by pressing icon

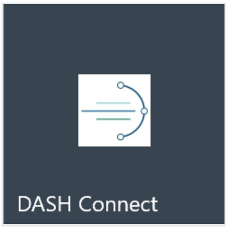

When the welcome screen shows, press the Open button

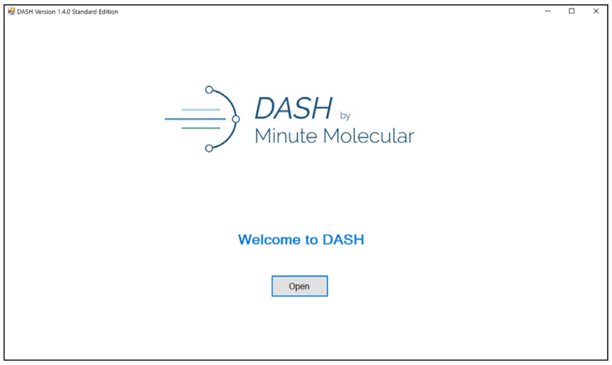

Touch the line with the serial number for the DASH Analyzer, which highlights it in blue.

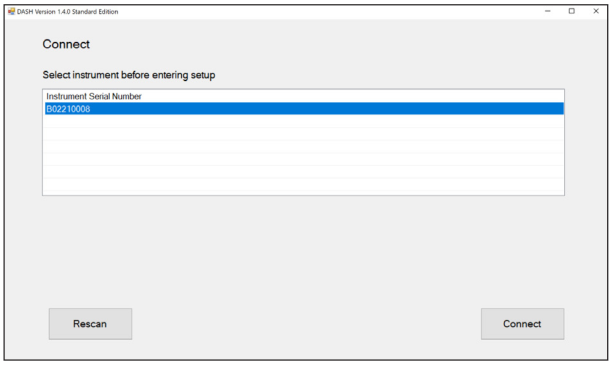

Press the Connect button, which downloads user, site, test results and other information from the DASH Analyzer

6.1.3 Home Menu Screen

The Home Menu screen displays

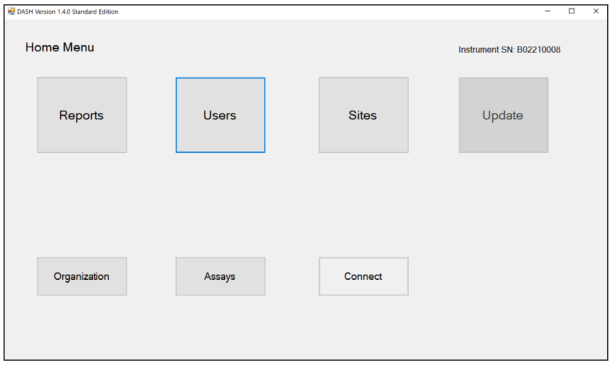

- The “Reports” button takes the user to the Reports screen.
- The “Users” button takes the user to the Users list screen.
- The “Sites” button takes the user to the Sites list screen.
- The “Update” button, when active, will update the connected Analyzer with any user or site information changes. It becomes active when a user changes the User content or Site information. Note: when the Analyzer is updated it will reboot for the changes to take effect.
- The “Organization” button takes the user to the Organization Information screen.
- The “Assays” button takes the user to the Assays list screen.
- The “Connect” button takes the user to the Connect screen to select the Analyzer to set up.
- The “Settings” button takes the user to a screen allowing the user to change the date and the time.

6.2 Display Reports

To review historical test results, press the “Reports” button on the Home Menu screen. The Reports List screen is shown below. It allows the user to view reports and export them. The columns of the data on the table include Specimen ID, Test Date, Result, LOT, User ID, REF, Assay Name, and Specimen Type.

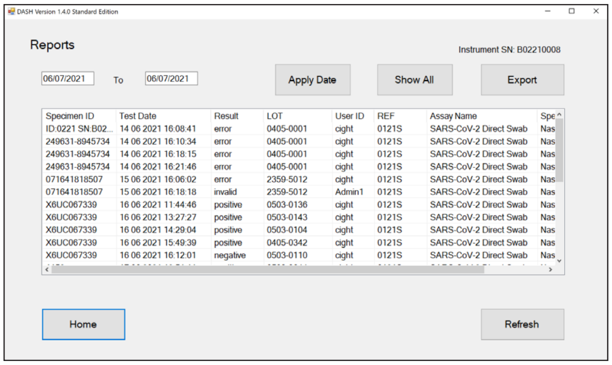

A description of each button’s function is given below.

- The “Home” Menu button takes the user back to the Home Menu screen.
- The “Apply Date” button runs a filter of reports based on the date range selected. By default, all reports are shown when entering the Reports List screen.
- The “Show All” button displays all reports.
- The “Export” button will prompt the user to enter a filename to save the shown reports list in CSV format.
- The “Refresh” button gets the most up-to-date reports data.

6.2.1 When clicking the “Export” button, the new window will pop up as below

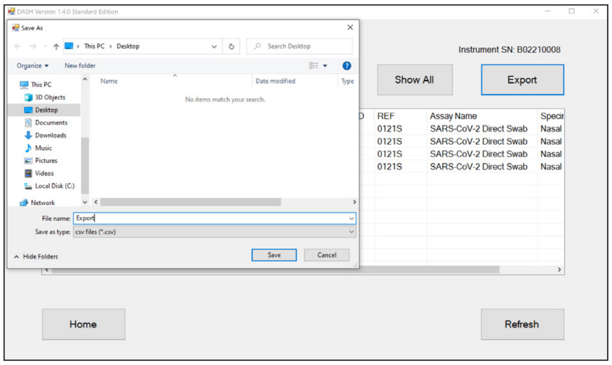

6.2.2 When changing the start date or end date and clicking the “Apply Date” button, the data on the reports list will adjust accordingly.

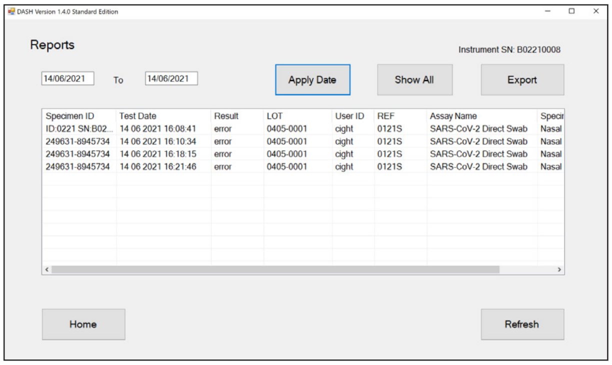

When the start date is greater than the end date, a pop-up window will appear.

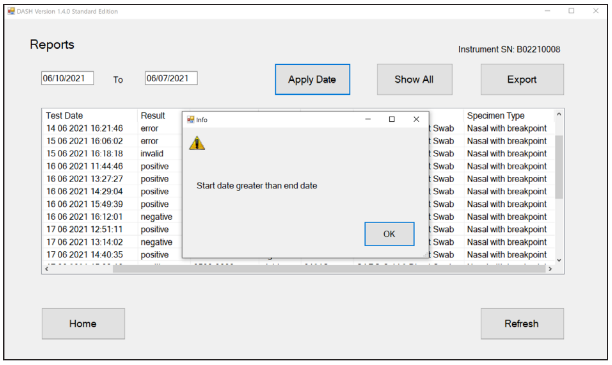

6.2.3 When the “Refresh” button is clicked, new reports will be added to the table.

6.3 Add a User

To add a user, press the “Users” button on the Home Menu Screen.

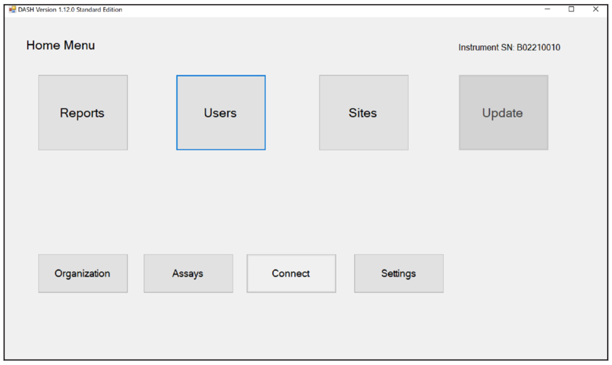

Then select the “Add User” button on the Users screen.

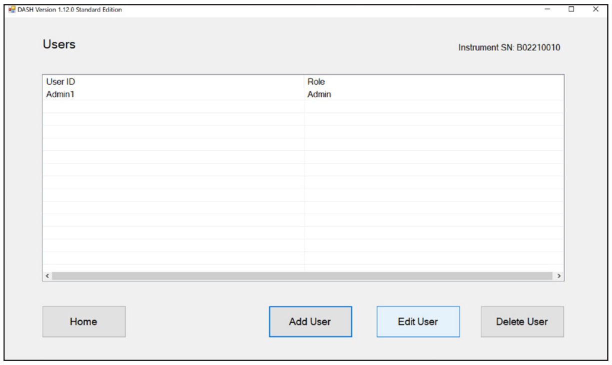

6.3.1 Assign a UserID and PIN

The Add User screen is shown below. It allows the user to add a new user.

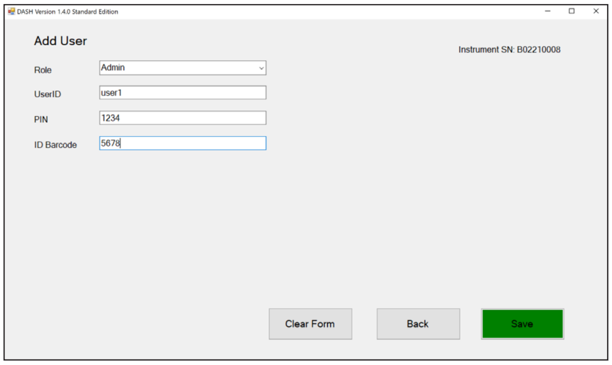

To add a user:

- Role: Select a role, ‘Admin’ or ‘User’, for the new user of the DASH Analyzer
- UserID: Enter a User ID that will be displayed on the DASH Analyzer.
- PIN: Assign a 4-digit, numeric code. The user will have to select their UserID and enter their 4-digit PIN when running a test on this DASH Analyzer. Please make notes of the UserIDs and PINs for later use on the DASH Analyzer. After Role, UserID, and PIN have been filled, the “Save” button will turn green.
- ID Barcode: This field is optional. If User will have a scannable barcode ID, then type the ID in this field.

6.3.2 Save your UserID and PIN

After clicking the “Save” button, a pop-up window will appear to notify the user that to update the Analyzer with changes go to the Home Menu screen.

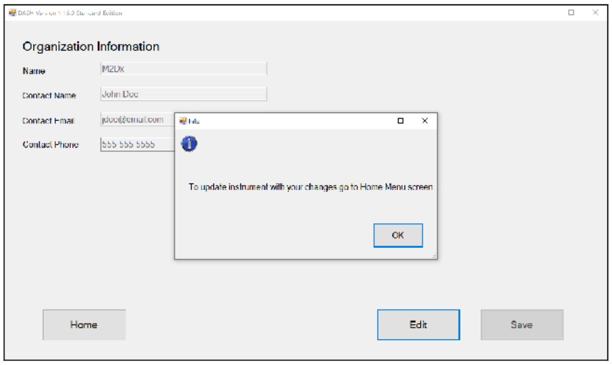

A description of each button’s function is given below.

- The “Back” button takes the user back to the User List screen.
- The “Save” button, when active, saves a new user to an internal database. Go to the Home Menu and click the “Update Analyzers” button to save information to the Analyzer.
- The “Clear Form” button clears the Add user form.

6.3.3 Edit User

The Edit User screen is shown below. It allows the user to modify certain user information. The PIN section only allows the user to enter 4 digits.

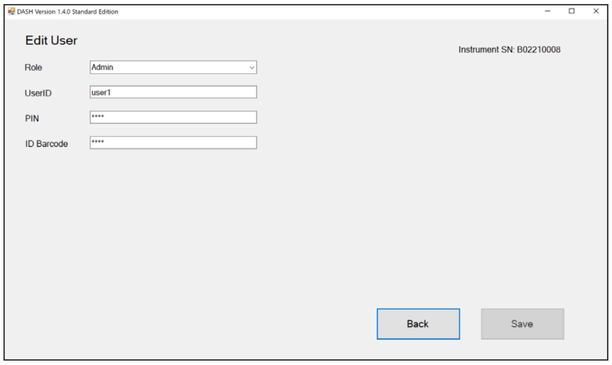

A description of each button's function is given below.

- The "Back" button takes the user back to the User List screen.
- The "Save" button, when active, saves any user changes to the internal database. Go to the Home Menu and click the "Update Analyzers" button to save information to the Analyzer.

6.3.4 Delete User

Select the user and click the "Delete User" button to delete the user. A pop-up window will ask to confirm the deletion.

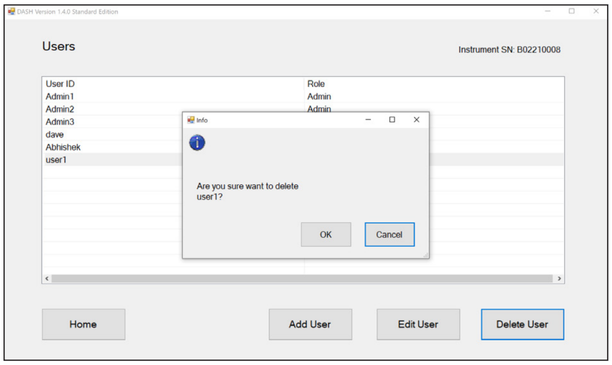

6.4 Assays List

The Assays List screen is shown below. It lists all the assays tested on the connected Analyzer. The "Home" button takes the user to the Home Menu screen.

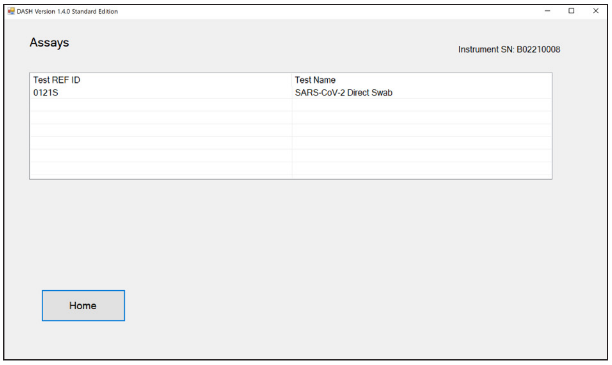

6.5 Site List

The Site List screen is shown below. It lists all the sites related to the connected Analyzer.

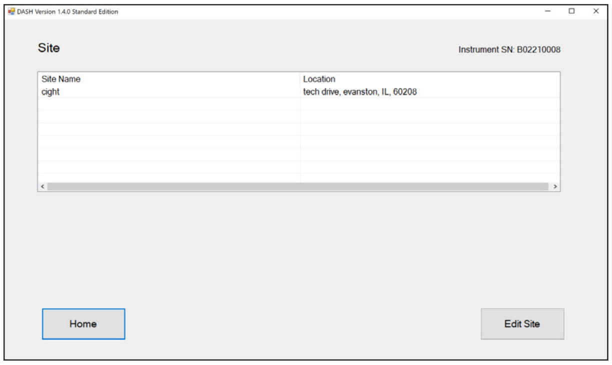

A description of each button's function is given below.

- The "Home" button takes the user to the Home Menu screen.
- The "Edit Site" button takes the user to the Edit Site screen.

6.5.1 Edit Site

When clicking the "Edit Site" button without selecting a site, the pop-up window appears.

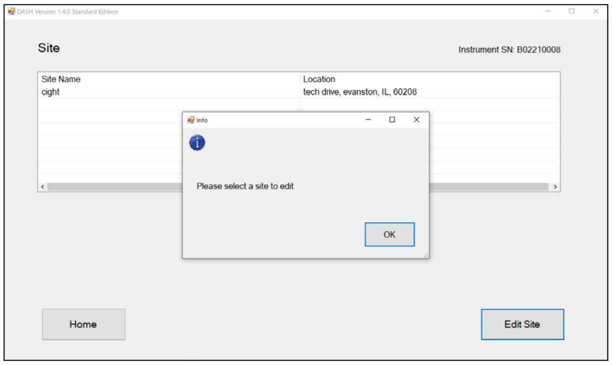

A description of each button's function is given below.

- The "Back" button takes the user back to the Site List screen.
- The "Save" button, when active, saves any site changes to the internal database. Go to the Home Menu and click the "Update Analyzers" button to save information to the Analyzer.

6.6 Organization

The Organization Information screen is shown below. It allows the user to modify organization information.

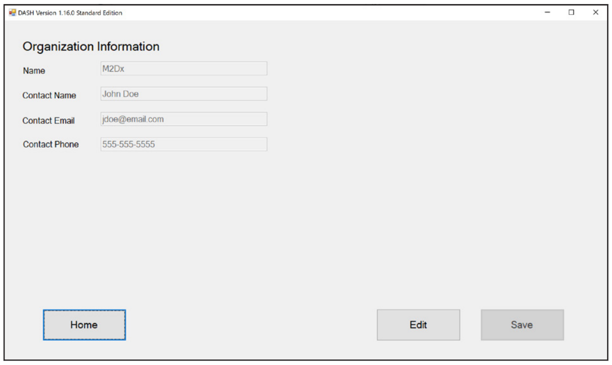

- The “Home” Menu button takes the user back to the Home Menu screen.
- The “Edit” button allows users to edit the organization information. Please note, to modify the location the user needs to use the Site screen.
- The “Save” button, when active, saves a new user to an internal database. Go to the Home Menu and click the Update Analyzers button to save information to the Analyzer.

6.6.1 When clicking the “Edit” button, the fields are able to be edited.

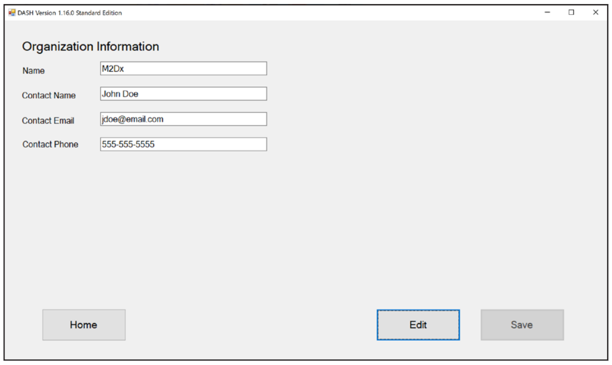

6.6.2 When clicking the “Save” button, a pop-up window will appear to notify the user to go back to the Home screen to update the Analyzer.

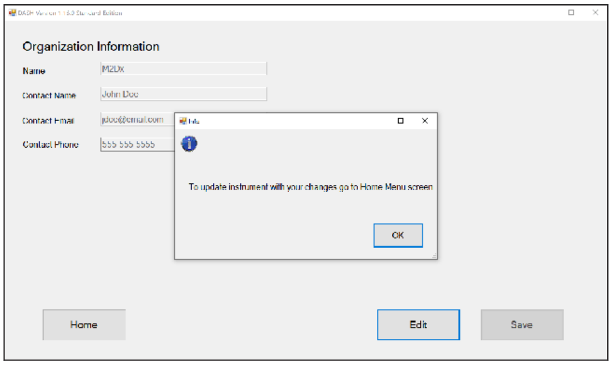

6.6.3 Click the “Home” button to go to the Home screen, the “Update” button will appear in green.

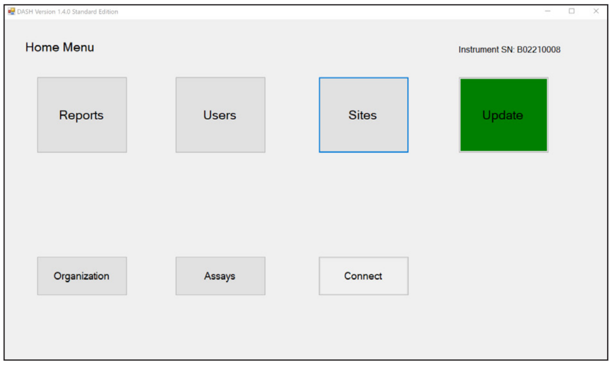

After clicking the “Update” button, the system will start to update the Analyzer.

6.7 Update Clock

If the DASH Analyzer has been unplugged for several days, it may be necessary to update the time. From the Home page, shown below, tap the “Settings” button.

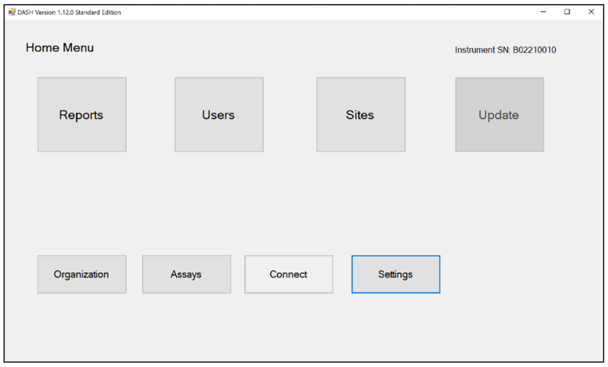

6.7.1 On the settings page, shown below, touch the up and down arrows to increase or decrease the seconds. The 3 numbers are hours, minutes, seconds.

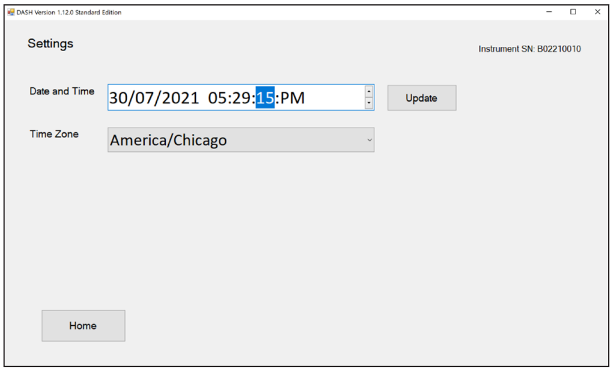

6.7.2 The hour and minutes values can be changed in a similar manner. Touch the value you want to update, and then the up and down arrows.

In the event that the date needs resetting, touch the day, month, or year, and enter the correct number. The order of the numbers are the date are day, month, year.

After you have the values you want to set, touch the Update button.

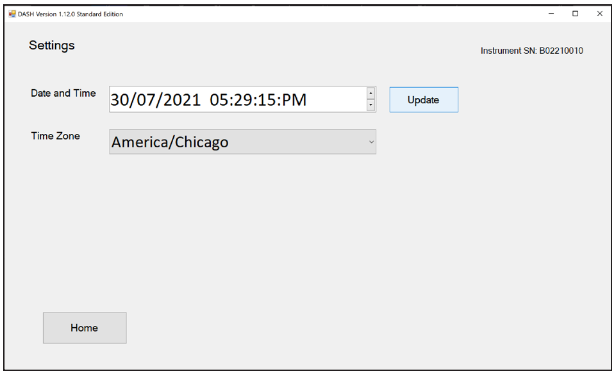

6.8 Error Screens

When attempting to update the Analyzer you may encounter an error pop-up screen. The screen will show the error reason and instructions on how to proceed. The following screens may pop-up when attempting to update the Analyzer.

6.8.1 User is logged into DASH Analyzer

The User Logged In error screen is displayed when the user is trying to update the Analyzer, and someone is logged into it. No one is to be logged in when updating the Analyzer.

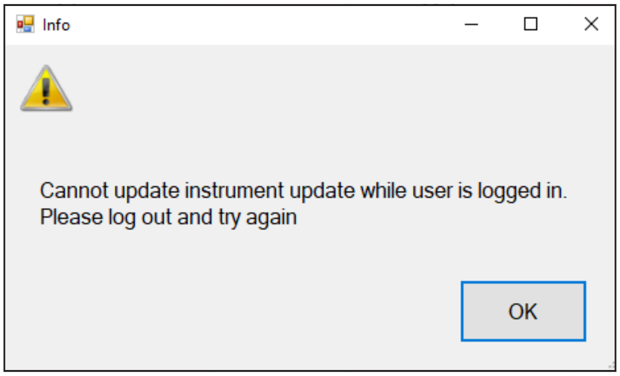

6.8.1 DASH Analyzer in Use

The Analyzer Busy error screen is displayed when the user is trying to update the DASH Analyzer while it is busy. The user must wait for the Analyzer to be idle before performing updates.

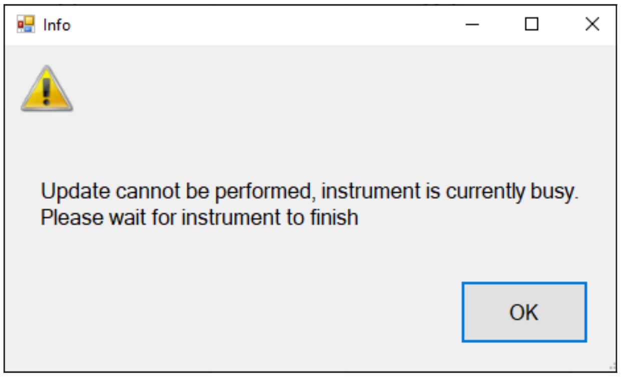

6.9 Exit

To exit the Tablet app, click the close tab on the upper right corner, and a pop-up window will appear to confirm.

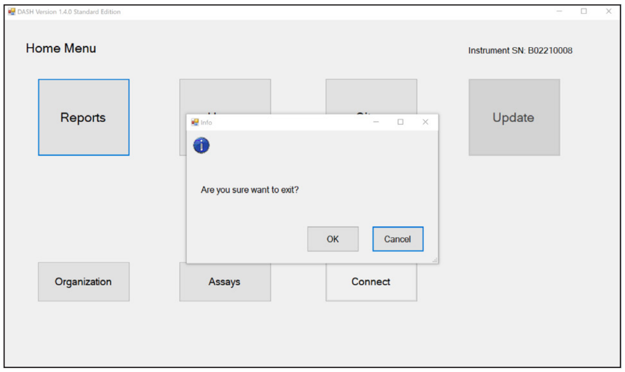

Limitations

- Performance of the DASH SARS-CoV-2/S Test has only been established in direct anterior nasal swab specimens. Use of the DASH SARS-CoV-2/S Test with other specimen types has not been assessed and performance characteristics are unknown.
- A false negative result may occur if a specimen is improperly collected, transported or handled. False negative results may also occur if inadequate numbers of organisms are present in the specimen.
- The performance of this test was established based on the evaluation of a limited number of clinical specimens. Clinical performance has not been established with all circulating variants but is anticipated to be reflective of the prevalent variants in circulation at the time and location of the clinical evaluation. Performance at the time of testing may vary depending on the variants circulating, including newly emerging strains of SARS-CoV-2 and their prevalence, which change over time.
- As with any molecular test, mutations within the target regions of the DASH SARS-CoV-2/S Test could affect primer and/or probe binding resulting in failure to detect the presence of virus, or the virus being detected less predictably.
- The performance of this test was validated using the procedures provided in this package insert only. Modifications to these procedures may alter the performance of the test.
- Negative results do not preclude SARS-CoV-2 infection and should not be used as the sole basis for treatment or other patient management decisions.
- Results from the DASH SARS-CoV-2/S Test should be correlated with the clinical history, epidemiological data, and other data available to the clinician evaluating the patient.
- DASH SARS-CoV-2/S Test has not been evaluated for patients receiving intranasally administer influenza vaccine.
- Biotin may interfere with the DASH SARS-CoV-2/S Test when present in anterior nasal swab specimens at concentrations >2.3 µg/mL.
- Performance has not been established in asymptomatic individuals.
- This test cannot rule out diseases caused by other bacterial or viral pathogens.
- The effect of interfering substances has been evaluated only for those listed within the labeling. Interference by substances other than those described may lead to erroneous results.
- The effect of homeopathic medications for respiratory symptoms on the assay performance was not tested.

Conditions of Authorization for Laboratories

The DASH SARS-CoV-2/S Test Letter of Authorization, along with the authorized Fact Sheet for Healthcare Providers, the authorized Fact Sheet for Patients and authorized labeling are available on the FDA website: **https://www.fda.gov/medical-devices/coronavirus-disease-2019-covid-19-emergency-use-authorizations-medical-devices/in-vitro-diagnostics-euas**

However, to assist clinical laboratories and Point of Care Settings using the DASH SARS-CoV-2/S Test (referred to in the Letter of Authorization as "Your Product"), the relevant Conditions of Authorization are listed below.

- Authorized laboratories[a] using your product must include with test result reports all authorized Fact Sheets. Under exigent circumstances, other appropriate methods for disseminating these Fact Sheets may be used, which may include mass media.
- Authorized laboratories using your product must use your product as outlined in the authorized labeling. Deviations from the authorized procedures, including the authorized instruments, authorized extraction methods, authorized clinical specimen types, authorized control materials, authorized other ancillary reagents and authorized materials required to use the DASH SARS-CoV-2/S Test are not permitted.
- Authorized laboratories that receive your product must notify the relevant public health authorities of their intent to run your product prior to initiating testing.
- Authorized laboratories using your product must have a process in place for reporting test results to healthcare providers and relevant public health authorities, as appropriate.
- Authorized laboratories using your product must collect information on the performance of your product and report to DMD/OHT7-OIR/OPEQ/CDRH (via email: CDRH-EUA Reporting@fda.hhs.gov) and Minute Molecular (+1 888-992-DASH or techsupport@m2dx.com) any suspected occurrence of false positive or false negative results and significant deviations from the established performance characteristics of the test of which they become aware.
- All operators using your product must be appropriately trained in performing and interpreting the results of your product, use appropriate personal protective equipment when handling this kit, and use your product in accordance with the authorized labeling.
- Minute Molecular, authorized distributors, and authorized laboratories using your product must ensure that any records associated with this EUA are maintained until otherwise notified by FDA. Such records will be made available to FDA for inspection upon request.

a. The letter of authorization refers to "authorized laboratories" as "laboratories certified under the Clinical Laboratory Improvement Amendments of 1988 (CLIA), 42 U.S.C. §263a, that meet the requirements to perform high, moderate or waived complexity tests. The DASH SARS CoV-2/S Test is authorized for use at the Point of Care (POC), i.e., in patient care settings operating under a CLIA Certificate of Waiver, Certificate of Compliance, or Certificate of Accreditation."

Performance Characteristics

Analytical Performance

Analytical Sensitivity (Limit of Detection)

Samples containing heat-inactivated SARS-CoV-2 virus (USA_WA1/2020, lot 70037779, BEI Resources, NR-52286) were tested at 50 copies/swab, 100 copies/swab, and 150 copies/swab in a range finding study by pipetting 20 µl of natural nasal swab clinical matrix contrived with heat inactivated SARS-CoV-2 virus onto nasal swab before testing in DASH SARS-CoV-2/S Test cartridge.

150 copies/swab was identified as the preliminary LoD. Twenty replicates of 150 copies/swab were tested and detected which verified that 150 copies/swab is the confirmed LoD. The results of the two studies are summarized below:

Table A. Limit of Detection Study Results

Inactivated SARS-CoV-2 Virus (USA_WA1/2020)	Copies/Swab Tested	DASH SARS-CoV-2/S Test Detected/Tested	DASH SARS-CoV-2/S Test Detected
Range-finding LoD Study	50	1/5	20%
	100	3/5	60%
	150	5/5	100%
Confirmatory LoD Study	150	20/20	100%

Analytical Reactivity (Inclusivity)

In silico analyses were performed using the RADx ROSALIND DXM online tool (https://radx.onramp.bio) to compare the DASH SARS-CoV-2/S Test primer and probe sequences for the N1 and N2 assays and the sequence specific capture probes to all SARS-CoV-2 sequences available in the ROSALIND database. The RT-qPCR oligonucleotide analysis was performed on February 17, 2022, and the capture probe analysis was performed on February 12, 2022. The sequences deposited in the previous 30 days before the analysis were also analyzed to report on recent trends in the data. The sequences used in the comparison are updated weekly from the GISAID Initiative by the ROSALIND tool. The potential impact of any oligonucleotide mismatch of a frequency greater than 0.1% was evaluated by calculating the predicted change in oligonucleotide Tm by comparing the oligonucleotide's Tm with and without the mutation in question using the nearest-neighbor two-state model. A sequence was considered at risk of reduced detection if the mismatch reduced the oligonucleotide's Tms at or below the PCR or the sequence specific capture hybridization temperature. 7,381,624 sequence were analyzed, and none of the sequences were predicted to have a risk of reduced detection. The results are presented in Tables below.

Table B. *In silico* analysis of SARS-CoV-2 N1 RT-PCR Oligonucleotides

	N1 Forward Primer		N1 Reverse Primer		N1 Probe	
	Frequency Across All Sequences	Frequency Past 30 Days (1/17/22-2/17/22)	Frequency Across All Sequences	Frequency Past 30 Days (1/17/22-2/17/22)	Frequency Across All Sequences	Frequency Past 30 Days (1/17/22-2/17/22)
Total Sequences Evaluated	7,381,624	57,966	7,381,624	57,799	7,381,624	58,007
1 Mismatch	1.03%	0.18%	0.36%	0.05%	16.74*	91.02%*
2 Mismatches	0%	0%	0%	0%	0.05%	0.27%
3 or More Mismatches	0%	0%	13.37%*	89.52%*	0%	0%

* The Omicron variant has a 7-nucleotide deletion in the 3' of the N1 reverse primer and a single mismatch to the N1 probe. The Tm of the truncated reverse primer and the probe are above that of the PCR hybridization temperature of the test.

Table C. *In silico* analysis of SARS-CoV-2 N2 RT-PCR Oligonucleotides						
	**N2 Forward Primer**		**N2 Reverse Primer**		**N2 Probe**	
	Frequency Across All Sequences	Frequency Past 30 Days (1/17/22-2/17/22)	Frequency Across All Sequences	Frequency Past 30 Days (1/17/22-2/17/22)	Frequency Across All Sequences	Frequency Past 30 Days (1/17/22-2/17/22)
Total Sequences Evaluated	7,381,624	57,866	7,381,624	57,846	7,381,624	57,662
1 Mismatch	0.73%	0.21%	0.54%	0.12%	1.0%	0.11%
2 Mismatches	0%	0%	0%	0%	0%	0%
3 or More Mismatches	0%	0%	0%	0%	0%	0%

Table D. *In silico* analysis of SARS-CoV-2 Sequence Specific Capture Oligonucleotides						
	**N1Cap2**		**N2Cap2**		**N3Cap1**	
	Frequency Across All Sequences	Frequency Past 30 Days (1/12/22-2/12/22)	Frequency Across All Sequences	Frequency Past 30 Days (1/12/22-2/12/22)	Frequency Across All Sequences	Frequency Past 30 Days (1/12/22-2/12/22)
Total Sequences Evaluated	7,210,955	198,498	7,210,955	197,684	7,210,955	196,832
1 Mismatch	0.25%	0.08%	0.88%	0.20%	0.53%	0.35%
2 Mismatches	0%	0.02%	0%	0%	0%	0%
3 or More Mismatches	10.3%*	85.0%*	0%	0%	0%	0%
* Omicron variant has 9-nucleotide deletion in the 3' of the N1Cap2 capture probe. The Tm of the truncated probe is above the annealing temperature of the probe hybridization to the viral RNA target.						

Delta (B.1.617.2, AY.1, AY.2, AY.3) and Omicron (B.1.1.529, BA.1, BA.2) variants are classified as circulating variants of concern (VOC). An in silico analysis of these variants was performed using the RADx ROSALIND DXM online tool (https://radx.onramp.bio) to compare the DASH SARS-CoV-2/S Test primer and probe sequences for the N1 and N2 assays and the sequence specific capture probes as of February 17, 2022. The predicted frequency of RT-qPCR oligonucleotide sequence mismatch is summarized in Table E. The sequence specific capture probes were also mapped on the sequences, and the predicted oligonucleotide sequence mismatch frequencies are summarized in Table F. No significant oligonucleotide mismatch with either RT-qPCR assay or sequence specific capture probes were observed with the Delta variant sequence analysis. One Omicron mutation that impacts both the N1Cap2 capture probe and the N1 reverse primer and a second mutation that impacts the N1 probe was observed. None of the resulting changes to oligonucleotide Tm resulted in a decrease to below their respective hybridization temperatures.

Table E. RT-qPCR Oligonucleotide Variants of Concern *in silico* Analysis Summ							
		Sum of % Frequency of Mismatches RT-qPCR Oligonucleotides*					
Variant of Concern	No. of Sequences Analyzed	N1 Forward	N1 Reverse	N1 probe	N2 Forward	N2 Reverse	N2 Probe
Delta B.1.617.2	165,962	0.51%	0.19%	1.52%	0.6%	0.77%	0.41%
Omicron B1.1.529 BA.1, BA.1.1, BA.2	1,099,394	0.07%	92.73%	94.79%	0.10%	0.05%	0.06%
* Any mismatch of frequency ≥ 0.1% was analyzed for Tm difference							

Table F. Capture Probe Variants of Concern in silico Analysis Summary				
		Sum of % Frequencies of Mismatches to DASH SARS-CoV-2/S Capture Probes*		
Variant of Concern	No. of Sequences Analyzed	N1 Cap1	N2Cap2	N3Cap1
Delta B.1.617.2	165,962	0.14%	0.31%	0.41%
Omicron B1.1.529 BA.1, BA.1.1, BA.2	1,099,394	72.83%	0.06%	0.06%
* Any mismatch of frequency ≥ 0.1% was analyzed for Tm difference				

Analytical Specificity/Exclusivity (Cross-Reactivity and Microbial Interference)

An *in silico* study for possible cross-reactions with related pathogens and normal or pathogenic flora that may be present in respiratory specimens (Table G) was conducted by mapping primers and probes in the DASH SARS-CoV-2/S Test individually to sequences downloaded from the NCBI database (September 01, 2021). The DASH SARS-CoV-2/S Test primers and probes target two distinct regions of the N gene, labeled as N1 and N2. The in silico analyses show no significant sequence similarities with either PCR target except SARS-CoV sequence. The N1 reverse primer has 90.91% identity, the N1 probe has 89.29% identity, the N2 forward primer has 100% identity and the N2 reverse primer has 92.59% identity. Direct wet testing of SARS-CoV in the DASH SARS-CoV-2/S Test was performed, and no cross-reactivity or assay interference was observed at the concentration tested (see below).

In addition to in silico analysis, wet testing was performed to evaluate the DASH SARS-CoV-2/S Test when in the presence of 32 viral and bacterial organisms. For the cross-reactivity, each organism was added to a DASH SARS-CoV-2/S Test cartridge with SARS-CoV-2 negative specimens in triplicate. No organism tested exhibited cross-reactivity of the DASH SARS-CoV-2/S Test at the concentration tested. The organisms, concentrations, and results are summarized in Table G:

Table G. Cross-reactivity Testing on the DASH SARS-CoV-2/S Test			
**Strain**	**Concentration tested**	**Units**	**Negative Samples (No. Valid Tests Negative for SARS-CoV-2)**
Human coronavirus HKU1*	1.00E+05	genomic copies/ml	3/3
Human coronavirus NL63*	1.00E+05	genomic copies/ml	3/3
Human coronavirus OC43*	1.00E+05	genomic copies/ml	3/3
Human coronavirus 229E*	1.00E+05	genomic copies/ml	3/3
Severe acute respiratory syndrome coronavirus-1 Urbani*	1.00E+05	genomic copies/ml	3/3
Middle East respiratory syndrome coronavirus*	1.00E+05	genomic copies/ml	3/3
Human adenovirus 2†	1.00E+05	$TCID_{50}$/ml	3/3
Human metapneumovirus*	2.50E-03	ng/µl	3/3
Human parainfluenza 1†	1.00E+05	$TCID^{50}$/ml	3/3
Human parainfluenza 2†	1.00E+05	TCID5^{0}/ml	3/3
Human parainfluenza 3†	1.00E+05	$TCID_{50}$/ml	3/3
Human parainfluenza 4a†	2.00E+03	$TCID_{50}$/ml	3/3
Influenza A H3N2†	1.00E+05	$CEID_{50}$/ml	3/3
Influenza A H1N1†	1.00E+05	$CEID_{50}$/ml	3/3
Influenza B†	1.00E+05	$CEID_{50}$/ml	3/3
Enterovirus 68*	2.50E-03	ng/µl	3/3
Human respiratory syncytial virus*	2.50E-03	ng/µl	3/3
Human rhinovirus 20 (strain 15-CV19)	1.00E+05	$TCID_{50}$/ml	3/3
Chlamydia pneumoniae†	1.00E+06	IFU/ml	3/3
Haemophilus influenzae†	1.00E+06	CFU/ml	3/3
Legionella pneumophilia†	1.00E+06	CFU/ml	3/3
*Mycobacterium tuberculosis**	1.00E+06	genomic copies/ml	3/3
Streptococcus pneumoniae†	1.00E+06	genomic copies/ml	3/3
*Streptococcus pyogenes**	1.00E+06	genomic copies/ml	3/3
*Bordetella pertussis**	1.00E+06	genomic copies/ml	3/3
*Mycoplasma pneumoniae**	1.00E+06	genomic copies/ml	3/3
Pneumocystis jirovecii†	1.00E+06	nuclei/ml	3/3
*Candida albicans**	1.00E+06	genomic copies/ml	3/3
*Pseudomonas aeruginosa**	1.00E+06	genomic copies/ml	3/3
Staphylococcus epidermis†	1.00E+06	genomic copies/ml	3/3
Streptococcus salivarius†	1.00E+06	genomic copies/ml	3/3
Pooled nasal wash	20%	% of specimen volume	3/3

*nucleic acid tested; †whole organisms tested

Analytical Specificity (Interfering Substances)

Potentially interfering endogenous and exogenous substances that may be encountered in upper respiratory specimens were evaluated by testing inactivated SARS-CoV-2 at a target concentration of 3X LoD in triplicate. It was determined that the volume of liquid the nasal swab can hold is 100 µl of specimen. Each substance was introduced into pooled negative human nasal matrix specimen at the concentration indicated in the table below in the specimen volume. No interference resulting in a negative result was observed in the presence of any of the substances at the concentrations shown in Table H. Biotin was found to interfere with the assay at levels above 2.3 µg/mL.

Table H. Potentially Interfering Endogenous and Exogenous Substances				
**Substance**	**Active Ingredient(s)**	**Concentration Tested**	**Units**	**# Positive Results / # Total Samples Tested**
Afrin® Original Nasal Spray	Oxymetazoline hydrochloride	5%	vol/vol	3/3
Chloraseptic® Throat Spray	Phenol, Glycerin	5%	vol/vol	3/3
Flonase®	Fluticasone Propionate	5%	vol/vol	3/3
Relenza	Zanamivir	0.3	mg/ml	3/3
Tobramycin	Tobramycin	1.0	mg/ml	3/3
Bactroban®	Mupirocin	10.0	mg/ml	3/3
Mucin	Purified mucin protein	0.5%	wt/vol	3/3
Biotin	Vitamin B-7	1.1	µg/ml	3/3
Biotin	Vitamin B-7	2.3	µg/ml	3/3
Biotin	Vitamin B-7	4.6	µg/ml	0/3*
Whole Blood	Blood (human)	10%	vol/vol	3/3
Viral Transport Medium	Transport medium	100%	vol/vol	3/3

*Invalid results obtained

Clinical Performance Evaluation

The clinical performance of the DASH SARS-CoV-2/S Test was evaluated using a total of 313 prospectively collected direct nasal swab clinical samples from individuals suspected of COVID-19 including those with signs and symptoms of a respiratory infection. Testing of clinical samples was performed with DASH SARS-CoV-2/S Test and a highly sensitive EUA molecular assay that the FDA has authorized for diagnostic testing of COVID-19.

The study results (Table I) demonstrated 95.9% positive percent agreement (PPA) and 98.5% negative percent agreement (NPA) between the DASH SARS-CoV-2/S Test on the DASH Analyzer and the comparator method. There were 6 discordant samples.

Table I. Clinical Performance Comparison with Comparator EUA Molecular Assay in Individuals Suspected of COVID-19

		**Comparator Results**		
		Positive	Negative	Total
DASH SARS-CoV-2/S Test	Positive	47	4[a]	51
	Negative	2[b]	260	262
	Total	49	264	313
PPA		95.9% (95% CI = 86.3% – 98.9%)		
NPA		98.5% (95% CI = 96.2% – 99.4%)		

[a] Four samples were positive in the DASH SARS-CoV-2/S Test, but negative in the comparator assay. All four of the samples were from known positive participants, and two had positive results in a tie breaker EUA RT-PCR test, one had an indeterminate result, and one had a negative result.

[b] Two samples were positive in the comparator assay, but negative in the DASH SARS-CoV-2/S Test. Both samples were low positives near or below the limit of detection for the comparator assay

Minute Molecular Diagnostics, Inc. Headquarters Location

1800 Sherman Avenue, Suite 504
Evanston, IL 60201, USA

Telephone: 1-888-992-DASH
www.m2dx.com

Technical Assistance

Before contacting Minute Molecular Technical Support, collect the following information:

- Product name
- Lot number
- Serial number of the instrument
- Error messages (if any)

Telephon: US + 1-888-992-DASH
Email: techservice@m2dx.com

Contact information for all Minute Molecular Technical Support offices is available on our website:
www.m2dx.com

References

https://www.cdc.gov/coronavirus/2019-nCoV/lab/guidelines-clinical-specimens.html

https://www.cdc.gov/coronavirus/2019-ncov/lab/guidelines-clinical-specimens.html

Table of Symbols

REF	Catalog Number
LOT	Lot
	Temperature Limitations
EUA	Emergency Use Authorization
	Consult Instruction for Use
Σ	Contains sufficient for X Determinations
	Manufacturer
	Use by or Expiration Date
Rx ONLY	Prescription use only device
IVD	In vitro diagnostic device
	Fragile, handle with care
	Caution
	Do not reuse

DASH SARS-CoV-2/S Test
Quick Reference Guide

DASH by Minute Molecular

Please refer to the Instructions for Use (IFU) for more detailed information.
Please refer the Package Insert for Specimen Collection and Handling.

IVD For use under Emergency Use Authorization (EUA) Only

For Direct Anterior Nasal Swab Specimens

For *in vitro* Diagnostic Use

For Rx Use Only

30°C / 15°C Store DASH Analyzer and Cartridges at room temperature (between 15˚C and 30˚C).

DO NOT use a Cartridge that is wet or has leaked.

DO NOT use a Cartridge whose top Covers are not intact.

DO NOT use a Cartridge that has been dropped.

DO NOT open Cartridge Cover #1, until you are ready to perform testing.

DO NOT open Cartridge Cover #2, until you are ready to insert Cartridge into the machine.

DO NOT flip Cartridge over after peeling off Cover.

DO NOT hold the Cartridge with the back end next to the Cover #2.

This product has not been FDA cleared or approved; but has been authorized for emergency use by FDA under an EUA for use by authorized laboratories.

This product has been authorized only for the detection of nucleic acid from SARS-CoV-2, not for any other viruses or pathogens.

The emergency use of this product is only authorized for the duration of the declaration that circumstances exist justifying the authorization of emergency use of in vitro diagnostics for detection and/or diagnosis of COVID-19 under Section 564(b)(1) of the Federal Food, Drug and Cosmetic Act, 21 U.S.C. § 360bbb-3 (b)(1), unless the declaration is terminated, or authorization is revoked sooner.

Materials Provided

DASH SARS-CoV-2/S Cartridge

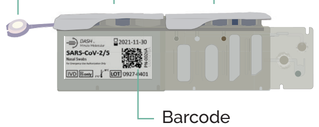

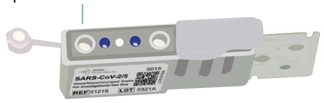

(Top View of Cartridge With Cover 1 Open)

Patient Swab

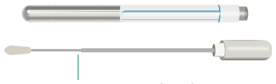

Required But Not Provided

Printer

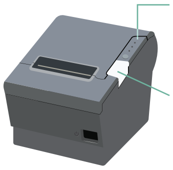

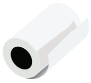

Printer Paper

DASH Analyzer

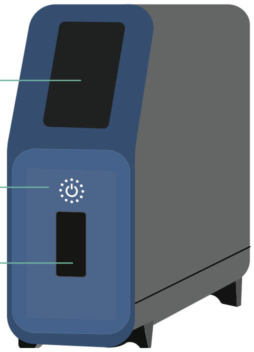

On Backside: (3) USB Connections; (1) Ethernet Port; and Power Connection

Barcode Scanner

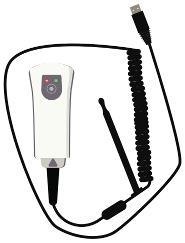

Before You Begin

- Samples should be tested immediately after sample collection.
- The environmental conditions for operating DASH SARS-CoV-2/S test are 15-30˚C, 20%-80% relative humidity.
- Follow the step-by-step instructions on the DASH Analyzer screen.

Video Tutorials

Unpacking	Setup	Running a Quality Control Sample	Running Patient Samples

TEST PROCEDURES begin on back

Minute Molecular Diagnostics, Inc.
1800 Sherman Avenue, Suite 504
Evanston, IL 60201, USA
1-888-992-DASH • techservice@m2dx.com

www.m2dx.com

PN-0206 REV 2022-03-09

Test Procedure: How to Test a Patient Specimen

DASH by Minute Molecular

1. Turn DASH Analyzer on and Login

When the DASH Analyzer is switched on, by pressing the Power Button, or after another User has logged out, the drop down list of usernames will be displayed.

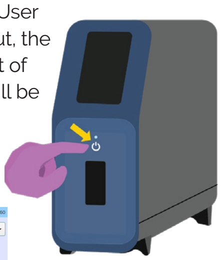

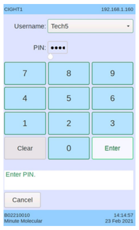

Select your username, and enter your 4-digit PIN and touch “Enter.”

2a. Scan specimen barcode

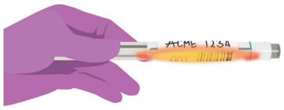

2b. Scan Cartridge barcode.
Run test immediately.

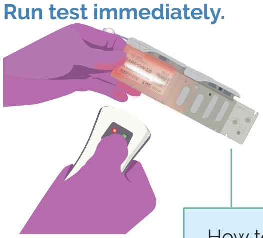

3. Follow instructions on the screen to prepare the specimen and Cartridge

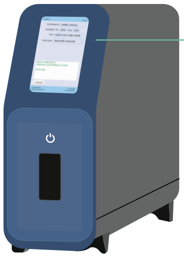

3a. Peel off Cover #1 ▶

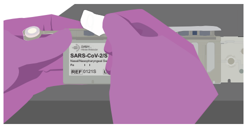

3b. Insert Swab into Specimen Chamber.

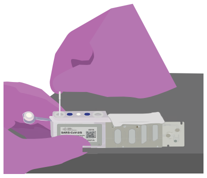

3c. Break off Swab stem and discard, and close Cartridge cap

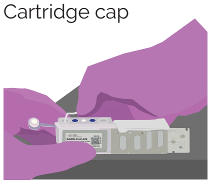

How to handle the Cartridge

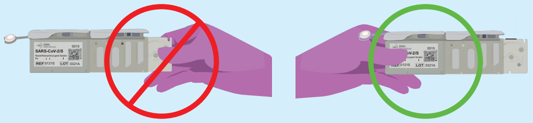

4. Peel off Cover #2.
Run test immediately.

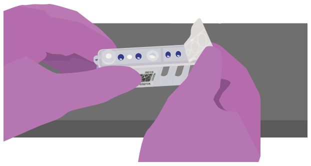

5. Insert the Cartridge ▼

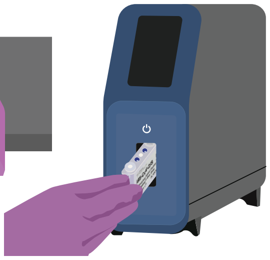

6. Test will run automatically. Light will indicate progress.

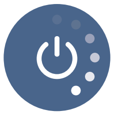

Power button with clock lights

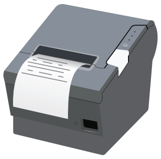

7a. After test is completed; the results will also print automatically.

7b. Touch “Open” to remove Cartridge.

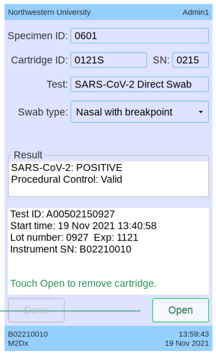

Result Interpretation

RESULT	INTERPRETATION
**SARS-CoV-2 NEGATIVE**	The 2019 novel coronavirus (SARS-CoV-2) target nucleic acids are not detected.
**SARS-CoV-2 POSITIVE**	The 2019 novel coronavirus (SARS-CoV-2) target nucleic acids are detected.
**INVALID**	Presence or absence of the 2019 novel coronavirus (SARS-CoV-2) target nucleic acids cannot be determined. Repeat test with a new nasal specimen and new cartridge.
**ERROR**	Presence or absence of the 2019 novel coronavirus (SARS-CoV-2) target nucleic acids cannot be determined. Repeat test with a new nasal specimen and new cartridge. Analyzer detected an error and aborted the current test.

Quality Controls: Positive and Negative Controls

Controls must be used to show that the Test is working properly. The DASH SARS-CoV-2/S Test Positive Control Swab (REF 0314P) and DASH SARS-CoV-2/S Test Negative Control Swab (REF 0315N) are available separately.

The DASH SARS-CoV-2/S Test Positive and Negative Control Swabs must be stored at room temperature. Controls are tested using the same procedure as for a patient sample, but instead of scanning the patient specimen barcode, scan the barcode on the Control Swab packaging.

It is recommended that external controls be tested at the frequency noted below.

- Each time a new lot or a new shipment of DASH SARS-CoV-2/S Test is received.
- Each time a new operator is performing the test.
- When the Analyzer is used for the first time.
- When the software is upgraded.
- When problems are suspected or identified.
- As deemed additionally necessary by internal quality control procedures, and in accordance with Local, State and Federal regulations or accreditation requirements.

If correct control results are not obtained, repeat the test using a new Control Swab, and a new test Cartridge.
If the control testing continues to fail, do not perform additional clinical specimen tests or report results.
If the positive or negative external controls fail, repeat with new external control and a new DASH SARS-CoV-2/S Test.
If the repeat test fails, please contact Minute Molecular Technical Support at 1-888-992-DASH or techservice@m2dx.com.

Minute Molecular Diagnostics, Inc. 1800 Sherman Avenue, Suite 504 Evanston, IL 60201, USA

Important information for DASH SARS-CoV-2/S Test users.

The kit may not include all of the labeling (e.g., paper copy).

This card is not a full set of instructions.

For the latest version of the labeling and instructions:

- Visit our website www.m2dx.com for access to labeling.
- Visit https://www.fda.gov/medical-devices/coronavirus-disease-2019-covid-19-emergency-use-authorizations-medical-devices/in-vitro-diagnostics-euas-molecular-diagnostic-tests-sars-cov-2
- Contact us at techservice@m2dx.com or 1-888-992- DASH for a free paper copy of the labeling and Instructions for Use.

This product has not been FDA cleared or approved; but has been authorized for emergency use by FDA under an EUA for use by authorized laboratories.

This product has been authorized only for the detection of nucleic acid from SARS-CoV-2, not for any other viruses or pathogens.

The emergency use of this product is only authorized for the duration of the declaration that circumstances exist justifying the authorization of emergency use of in vitro diagnostics for detection and/or diagnosis of COVID-19 under Section 564(b)(1) of the Federal Food, Drug and Cosmetic Act, 21 U.S.C. § 360bbb-3 (b)(1), unless the declaration is terminated, or authorization is revoked sooner.

IVD Rx ONLY For use under Emergency Use Authorization (EUA) Only
