# Supplementary material for: Clinical evaluation of the Diagnostic Analyzer for Selective Hybridization (DASH): A point-of-care PCR test for rapid detection of SARS-CoV-2 infection
Source: PLoS One. 2022 Jun 16;17(6):e0270060. doi: 10.1371/journal.pone.0270060 (PMC9202852; doi:10.1371/journal.pone.0270060)
Supplement: Attachment — Submitted filename: PLOS ONE DASH Manuscript Response to Reviewers.docx [file pone.0270060.s004.docx]

**Response to Reviewers: Editor/Reviewer Points (***italics***) and** Rebuttal

For each rebuttal, references to Page and Line numbers are for the ‘Revised Manuscript with Tracked Changes’ document.

**Academic Editor**

*One of the main concerns which I shared is the lack of information about DASH.  Any background, principle, reference(s) that could help readers understand the mystery of the blue box?*

Rebuttal: Since initial submission of this manuscript, DASH SARS-CoV-2 POC fast PCR test received approval from U.S. FDA for Emergency Use Authorization (EUA) on March 15, 2022. The principles of the procedure are in the Instructions For Use (IFU) published on the FDA website (<https://www.fda.gov/media/156979/download>). This has now been provided as a supplement (S1) and references 24 to 26 were inserted in several sections of the manuscript so that readers can get a better understanding of this new technology and how it operates.

*Specific comments:*

*1. Suggest combine Fig. 1 & 2 into one*

Rebuttal: This is a terrific suggestion; we have now combined Fig 1 and Fig 2 into a single Fig 1 with panels A and B. The manuscript text and figure legends have been updated to reflect this.

*2. Results, first paragraph, line 5 (page 8):  Suggest change "... exposure to someone COVID-19" to "...exposure to someone with COVID-19"*

Rebuttal: Thank you for this suggested change, we have made this adjustment at line 200 on page 8 of the manuscript.

*3. Discussion, page 15:   Please rephrase the last sentence for clarity.*

Rebuttal: We have modified this part of the manuscript on pages 16-17 and lines 334-351 of the manuscript to provide greater clarity and response to Review #1, comment 2, as below.

*4. Discussion, page 16, second paragraph:  Regarding the dynamic detection range, has this being done by the manufacture or during CLIA certification?*

Rebuttal: Range-finding limit of detection (LoD) studies were performed by Minute Molecular and can be found on page 32 of the IFU under “Performance Characteristics” (see S1). In this section of the Discussion, we were referring to the “course” of infection within an individual rather than technical dynamic range of the device. The wording was changed to reflect this at line 353 on page 17.

**Reviewer #1**

*1. The DASH device needs to be described in more detail*

Rebuttal: We have provided more details of the DASH technology through a supplement PDF of the DASH IFU (S1) and we have provided references (numbers 24 to 26) — published on the FDA website (<https://www.fda.gov/media/156979/download>) as part of the FDA EUA approval.

*2. The comparison of raw Ct-values is interesting (fig. 4/5), but the authors need to measure virus preparation standards with known virus concentrations to compare semi quantitative virus concentrations rather than the Ct values which depend on the specific instruments. It is nowadays necessary to know, whether the virus load is below 10exp6. See "SARS-CoV-2 screening in patients in need of urgent inpatient treatment in the Emergency Department (ED) by digitally integrated point-of-care PCR: a clinical cohort study. by Möckel M, Bolanaki M, Hofmann J, Stein A, Hitzek J, Holert F, Fischer-Rosinský A, Slagman A.*

*Diagn Microbiol Infect Dis. 2022 Jan 16;102(4):115637. doi: 10.1016/j.diagmicrobio.2022.115637." for details.*

Rebuttal: We agree with the reviewer on this point. Our goal of this analysis was to understand general trends in comparison of Ct values generated by these technologies with limitations on how virus is detected using specific PCR platforms/instruments. The manuscript was updated on pages 16-17 and lines 334-351 of the discussion to address this issue and the reference listed above by the reviewer was added.

*3. Part of the comparison needs to be the sample management and handling at the point of care. The authors should give more details here. Are there regulatory issues and safety requirements to process potentially infectious samples?*

Rebuttal: We provided details of how each nasal swab sample was collected and handled at the point care in the Methods on pages 5-6 and lines 105-132 of the manuscript. As per this reviewers suggestions, we have provided additional details on handling of specimens including safety precautions that were in concordance with institutional infection control policies.

*4. The data availability statement is incomplete. There are no raw data available and also no source for it.*

Rebuttal: We have now provided a de-identified raw dataset as a CSV file and completed the data availability statement.

*5. The authors provide data on frozen sample stability. They should in addition discuss as a limitation that the measurement from frozen samples is not comparable with acute sample measurement.*

Rebuttal: We agree and have added this to our limitations section of the Discussion on page 17 at lines 354-357 of the manuscript.

*6. The authors used anterior nasal swabs which are less sensitive compared to deep nasophayryngeal swabs. This should be discussed.*

Rebuttal: We agree and have added a section to the limitations paragraph of the Discussion on this topic on page 18, and lines 373-380 of the manuscript.

*7. The participants did not get results from the experimental device. Therefore, no safety consideration could be assessed. I believe that the results are therefore preliminary and an additional study with the experimental device for clinical use and backup comparison with a reference method is necessary.*

Rebuttal: The standard approach from FDA for clinical studies on experimental novel PCR diagnostics for SARS-CoV-2 is to only give results to participants on the standard FDA EUA approved diagnostic PCR test; which is what we did in this study. We do not believe there are any public health or safety issues as the participants were promptly (within 24 hours) given results of their Cepheid Xpert SARS-CoV-2 PCR test. Now that DASH has received FDA EUA approval, we are planning for additional clinical validation studies where the participants will be given DASH results. We briefly added text to the discussion on page 17 and lines 357-360 regarding this limitation.

*8. I do not understand the figure 3. Is this a simulation? The authors did not gather data from different populations. I think this figure is superfluous.*

Rebuttal: We respectfully disagree with reviewer #1 and believe that Figure 3 (now Fig 2) is not superfluous as it graphically addresses the concept of how SARS-CoV-2 diagnostic test performance depends on population prevalence of infection. As described in the Methods on page 7 and lines 171-175 of the manuscript, we calculated PPV and NPV using Bayes theorem at 8%, but also estimated these parameters for a range of prevalence from 1 to 30% with confidence intervals for predictive values generated using 1000 bootstrap replicates.

*9. The authors should consequently use "SARS-CoV-2" if they address the virus and "COVID-19" only if they address the disease. Nobody is "COVID-19 positive" (see abstract for example)*

Rebuttal: We have made adjustments throughout the manuscript to consistently use “SARS-CoV-2” when referring to the virus and “COVID-19” when referring to the disease.

**Reviewer #2**

*Introduction*

*1. A reference is needed for the following text: “DASH utilizes rapid PCR and microfluidic fabrication technology with uniquely designed cartridges and an analyzer that are easy to use, do not require technical expertise or specialized training, and produce results in approximately 15 minutes. In laboratory testing, DASH was found to have analytic sensitivity at the same level as laboratory-based PCR with the ability to detect at least 100 copies/mL of SARS-CoV-2 RNA”.*

Rebuttal: We have provided references (numbers 24-26) for the DASH technology and DASH IFU recently published on the FDA website and company website as part of the DASH EUA approval.

*2. Please add more information on the DASH SARS-CoV-2 assay, such as the target genes, PCR cycles, etc.*

Rebuttal: The target genes are 2 PCR targets in the N gene (N1 and N2) and the number of PCR cycles is 40. We have provided these details on page 3 and lines 66-67 long with supplemental materials and a reference to the DASH IFU published on the FDA website.

*Methods*

*1. Page 5, viral transport media (VTM): Please add the volume of VTM and brand name (including company information).*

Rebuttal: The VTM used was MicroTest M4RT Transport made by Remel with volume of 3.0 mL. This information was added under the Methods on page 5 at lines 118-119.

*2. What kind of swabs were used in the current study?*

Rebuttal: Both nasal swabs obtained from participants were Puritan Ultra 6” Sterile Elongated Flock Swab with Plastic Handle & Dry Transport Tube (SKU #25-3606-U BT). This information was added to the Methods of the manuscript on page 5 at lines 109-110.

*Methods/Results/Discussion*

*1. How about invalid results in this study? A DASH re-test cannot be performed with the initially obtained samples. What do technicians do if the test results are not obtained properly due to invalidity? When emphasizing the merits of DASH in the Discussion, you should also describe the demerits.*

Rebuttal: As mentioned on page 8 at line 196 and page 16 line 325, we only had 2 invalid results (0.6%) and data from these two participants were removed from the final analysis since we were unable to compare to the standard PCR result. For this study, we provided the participant their standard SARS-CoV-2 PCR result as we did for all other participants as per the standard of care. Now that the DASH technology has received FDA EUA and can be used in clinical care, we would recommend that in the event of an invalid DASH result, the technician would inform the provider team who would need to collect another anterior nasal swab from the patient and run it again on DASH. This is a downside to DASH and other POC diagnostics without residual specimens/VTM after testing. We added this “demerit” to our Discussion on page 16 at lines 325-328.

*2. The current evaluation was only performed with anterior nasal samples. If nasopharyngeal samples or saliva are used for the evaluation, false-positive results might be obtained. You should mention that current investigation was only performed with anterior nasal samples.*

Rebuttal: We agree and have added a paragraph on this topic of nasal versus nasopharyngeal swabs to our Discussion on page 18 at lines 373-380 of the manuscript.

*3. In this study, most of the positive samples were obtained from patients with preceding positive results for SARS-CoV-2 according to other molecular assays. The current results should be validated in a further study. You should mention the limitations in the Discussion.*

Rebuttal: We will have added this detail to our limitations paragraph of the Discussion on page 17 at lines 360-361.
